# On the Computational Design of Azobenzene-Based Multi-State Photoswitches

**DOI:** 10.3390/ijms23158690

**Published:** 2022-08-04

**Authors:** Miquel Moreno, José M. Lluch, Ricard Gelabert

**Affiliations:** 1 Departament de Química, Universitat Autònoma de Barcelona, 08193 Bellaterra, Barcelona, Spain; 2 Institut de Biotecnologia i de Biomedicina (IBB), Universitat Autònoma de Barcelona, 08193 Bellaterra, Barcelona, Spain

**Keywords:** molecular photoswitches, azobenzene dimers, theoretical photochemistry, absorption spectra

## Abstract

In order to theoretically design multi-state photoswitches with specific properties, an exhaustive computational study is first carried out for an azobenzene dimer that has been recently synthesized and experimentally studied. This study allows for a full comprehension of the factors that govern the photoactivated isomerization processes of these molecules so to provide a conceptual/computational protocol that can be applied to generic multi-state photoswitches. From this knowledge a new dimer with a similar chemical design is designed and also fully characterized. Our theoretical calculations predict that the new dimer proposed is one step further in the quest for a double photoswitch, where the four metastable isomers could be selectively interconverted through the use of different irradiation sequences.

## 1. Introduction

Molecular photoswitches are nowadays at the heart of a large number of very hot topics in research within a broad range of sciences, ranging from nanotechnology to pharmacology all the way through catalysis and biochemistry [1,2,3,4,5,6,7,8,9]. The most frequently used photoswitches are azobenzenes (ABs) as they are easy to synthesize, are highly reversible and photostable and have quite different molecular structures between the photoconvertible cis and trans isomers [2,5,10]. Moreover, the photochemical properties of ABs can be largely modulated by the introduction of different substituents on the benzene rings [11,12,13].

The traditionally used molecular photoswitches involve a binary (on/off) pair corresponding to the two photoconvertible isomers (usually cis/trans or Z/E). Recently, interest has turned to the design of multi-photochromic compounds that contain two or more switching centers usually linked by covalent bonds [14,15,16]. In the simpler case of two such centers, four states can be interchanged by means of light (or another external stimulus). This allows for more complex photochemical processes including the possibility to design molecular motors that perform unidirectional cycles [17,18,19]. Not surprisingly, ABs are the most commonly used units in the design of such multi-photochromic systems [20,21,22,23,24,25,26,27,28,29,30].

The first and simplest double photoswitch analyzed was a bis(azo) compound where two azobenzene units shared a central phenyl ring. Such a conjugated system does not allow for selectively addressing each AB unit, but the introduction of different substituents at each phenyl group leads to four potentially stable isomeric forms [20,21,22,23]. In order to have two (or more) AB units that could be “orthogonally” photoexcited (i.e., where each AB unit could be independently and selectively activated), a more complex design is necessary. One strategy towards this goal involves incorporating AB units in macrocyclic arrangements, resulting in azobenzophanes [24,25]. More recently, interest has focused on the design of star-like triple azobenzene units linked to a central core, as the resulting tripodal design affords a wide range of applications [26,27,28,29]. Another strategy involves the synthesis of azobenzene dimers with bulk substituent groups so that both moieties are almost orthogonal due to steric repulsions [30,31,32,33].

In some of the cited references on multi-azobenzenes, theoretical calculations are also performed [20,21,22,23,24,26,27,28,29,33], though they are usually restricted to the ground electronic state. Slavov et al. [23] reproduce the absorption spectra of unsubstituted bisazobenzenes with a common phenyl ring, obtaining good accordance with the experimental results. The photoinduced E → Z isomerization of these azobenzenes is also studied by Floss and Saalfrank through a surface-hopping molecular dynamics study [22] demonstrating that, depending on the connectivity pattern of the two azo groups, they may act as uncoupled units or else present a cooperative behavior. Bléger et al. [33] carry out extensive ab initio calculations devoted to obtaining the absorption spectrum and perform a quite comprehensive theoretical analysis of the potential energy surface of the ground electronic state of the unsubstituted bisazobenzene with the two moieties uncoupled. On the theoretical side, the most studied multiazobenzene systems are the systems with three azobenzene units linked to a central core [26,27,28]. The pure theoretical work by Yang et al. [27] points to a decoupling of the three azo groups so that introducing different substituents at each AB branch should lead to an effective triple independent photoswitch.

Given that previous theoretical calculations have been restricted to investigating specific systems already synthesized and experimentally studied, in this work we propose a comprehensive analysis of the factors that govern the photochemistry of a generic azobenzene-based multi-state photoswitch. As a first but relevant step towards this goal, we will consider azobenzene dimers with different substituents so that four E/Z isomers are potentially stable. The ideal goal would be to find systems where all the interconversions (up to six in a dimer) could be triggered by different sequences of irradiation, so each isomer could be selectively addressed.

## 2. Results and Discussion

**Scheme 1:**
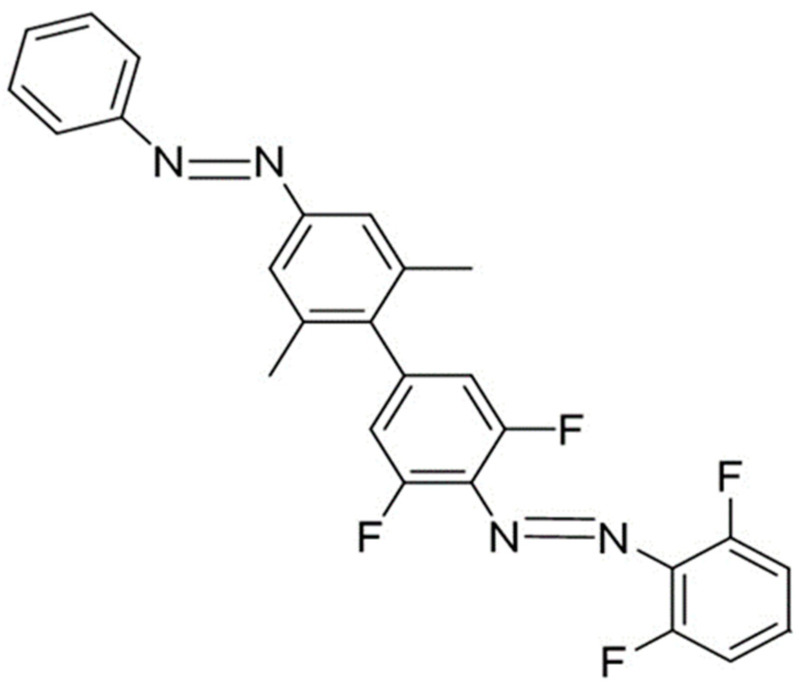
Molecular structure of dimer 1 in its most stable (EE) form.

The first step will be to assess the reliability of our methodology, comparing with known experimental data. For this we take the bisazobenzene synthesized and experimentally analyzed by Zhao et al. [30] as a model to analyze the factors that govern the photochemistry of such a dimers. Scheme 1 shows the molecular structure of this system, labelled dimer 1 from now on. It consists of an unsubstituted azobenzene covalently linked to a full ortho-fluorated one. The two methyl groups at one end of the benzenic ring are introduced to keep the two units in quasi-orthogonal planes.

The four E/Z isomers expected to be stable for this system will be labeled EE, EZ_F_, ZE_F_ and ZZ. A subindex F is used when the E/Z state of the azobenzene unit (first letter) and the fluoroazobenzene part (second letter) are different.

Table 1 presents the energies of the lowest excited electronic states for the four stable E/Z isomers of dimer 1. The excited states are calculated at the geometry of the minima in the ground state (vertical excitations) so that the calculated excitation wavelengths should approximately match the peaks of the bands in the experimental absorption spectra. Only excited states with an absorption wavelength above 250 nm are considered in Table 1 as this is the approximate lower limit of the experimentally obtained spectra. f values report the oscillator strength of the transition from the ground electronic state. A value below 0.05 usually means that the S_0_ → S_n_ transition is forbidden, so it should be barely visible in the actual spectra. The last column in Table 1 indicates the main contributions to the excitation. Capital H and L letters stand for HOMO and LUMO, respectively.

**Figure 1:**
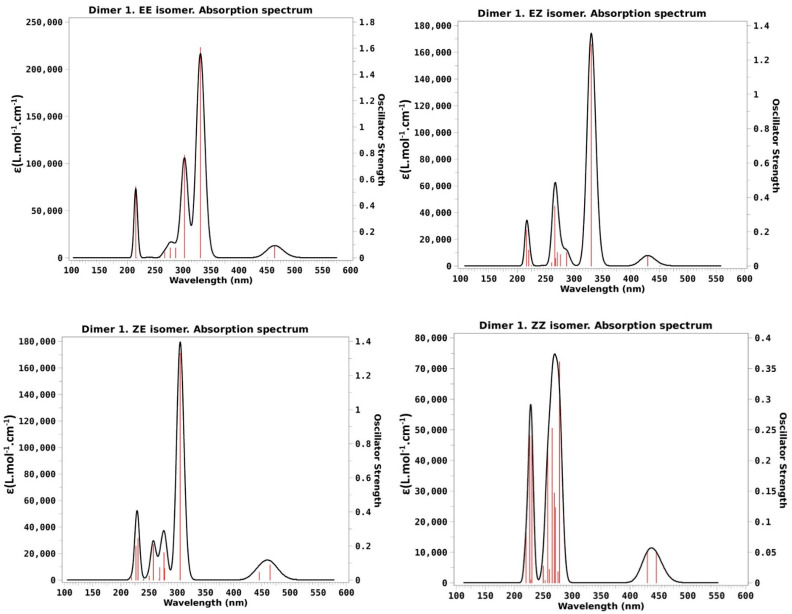
Computed absorption spectra of dimer 1 in acetonitrile at the optimized ground-state geometries of the four stable isomers of dimer 1. Vertical red lines indicate the position of the vertical transitions while the black contour corresponds to the convolution with a Lorentzian function depicted in order to theoretically reproduce the experimental absorption spectrum.

The first use of the numerical results presented in Table 1 is that they allow us to compute the absorption spectra of the four stable isomeric forms of dimer 1, as shown in Figure 1. The spectra, obtained using the GaussView program [34], are convoluted with a Lorentzian function with full-width-at-half-maximum of 0.1 eV. At first sight, comparison of Figure 1 with the experimental absorption spectra as presented in Figure 2 of [30] reveals that our calculation is accurate enough. It is to be considered that bands depicted in Figure 1 below 250 nm are beyond the experimental range.

Using the data reported in Table 1, a more detailed comparison of the theoretical and experimental spectra can be made to actually assess the feasibility of our computational strategy.

- EE isomer: Experimentally this is the only stable isomer in the absence of irradiation. The main feature is a huge broad band that extends between 280 and 390 nm, with a maximum at approximately 340 nm. An additional, much tidier, but also broad band is seen at 400–500 nm. Our theoretical calculation reveals that the larger band includes both the transitions to S_3_ and S_4_ that have large f values 1.61 and 0.78 in our calculation, whereas the less intense band at lower energy will correspond to the transition to S_1_ calculated at 464 nm that has a small, but not negligible, f value of 0.10. The close transition to S_2_ calculated at 464 nm has an oscillator strength of 0.10, a small though not negligible value.
- EZ_F_ isomer: Experimentally this isomer is the prevalent one (79%) when the original dimer is irradiated at >500 nm, so we can compare the results in Table 1 with the photostationary state (PSS) spectra after irradiation at this wavelength. In comparison with the EE case just discussed, there are very few differences in the position of the absorption bands. Intensities are different but this may be partially due to the fact that we are now analyzing a mix of different isomers. At any rate the calculations of the excited states of EZ_F_ isomer disclose again the presence of three allowed transitions with f > 0.1: The more intense one to S_3_ at 330 nm (f = 1.29) would make for the higher intensity experimental band with the little help of transition to S_4_ calculated at 287 nm with f = 0.08. The lower energy will correspond to the S_2_ transition predicted at 430 nm with a very low but non-zero f value of 0.06.
- ZE_F_ isomer: This structure is not obtained from direct irradiation so that now there is no experimental data to compare with. In any case the calculations predict again rather small differences with respect to the previous discussed cases: The most likely transition is to S_3_, calculated at 305 nm with f = 1.33, while the lower energy transitions to S_1_ and S_2_ are predicted to peak at 465 and 445 nm, respectively, but have, as in the previous cases, quite small intensities (f < 0.1).
- ZZ isomer: This species is dominant (74%) when the original dimer is photoexcited at 350 nm. Now, the obtained spectrum is clearly different from the previous cases just discussed: The larger band is now seen at noticeably shorter wavelengths that almost reach the end of the experimental available range (λ < 300 nm). The less intense band at lower energies is also slightly blue-shifted with a maximum slightly above the 400 nm mark. Our results are in quite nice agreement with these facts as the more intense band is the one to S_3_ calculated at 278 nm while the two lower transitions to S_1_ and S_2_ have very low f values (0.05) and are predicted to appear at 430 and 445 nm. Another noticeable fact of the ZZ absorption spectrum presented in [30] is the lower intensity of the high energy band as compared with the corresponding one of the other two obtained isomers. This is also in nice agreement with our theoretical results as the higher oscillator strength of the ZZ isomer just reaches the 0.36 value for the transition to S_3_, well below the corresponding maxima of the EE (1.61) and EZ_F_ (1.29) structures, as seen in Table 1.

**Figure 2:**
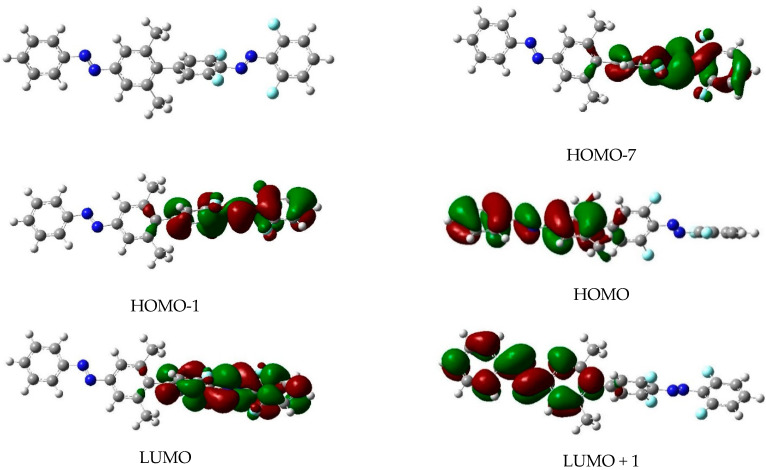
Shape of the molecular orbitals involved in the relevant excited electronic states of the EE isomer of dimer 1. The drawings correspond to an iso-level of 0.02 a.u. of electronic density. The left top corner of the figure depicts the molecular structure of the EE isomer.

A different question not directly related to the electronic absorption spectra but crucial in the design of dimer 1 is the orthogonality of the electronic excitations. That is: dimer 1 was synthesized in order to be able to selectively excite each one of the two azobenzene units. This is the reason for the presence of the two ortho-methyl groups, as in one of the benzenic rings: so that the π systems of both moieties remain in almost perpendicular planes. Our calculation reveals that, concerning the geometries, this goal is accomplished (See Figure 2, top left). To address this question more systematically, in the last column of Table 1, a letter H or F is used to indicate whether the electronic excitation is located in the parent azobenzene unit (H) or in the ortho-fluorinated one (F). As the two AB moieties are almost orthogonal in the most stable EE form, we note that all the electronic excitations of the EE isomer are fully located in one AB unit and so can be safely labeled as H or F.

This separation is also clearly seen in Figure 2 where the orbitals that are involved in the different excited electronic states described in Table 1 are drawn. The shape of the molecular orbitals depicted in Figure 2 confirms that the two lowest excited electronic states, S_1_ and S_2_, come from excitations from orbitals that might be labeled as n (as they are not π) to a clear π*-type orbital (the asterisk appears as the arriving orbital is formally empty in the ground electronic state), so that these transitions are formally forbidden (low value of f), whereas the next two states, S_3_ and S_4_, come from excitations between π orbitals (and are formally allowed). The shape of the orbitals seen in Figure 2 also confirms the orthogonality of the different electronic excitations so that all the orbitals are located in one side of the dimer. For the other three isomers, the analysis of the molecular orbitals is more problematic as the planarity of the benzenic rings is partially lost in the Z isomers due to steric repulsions. At any rate the orthogonality of the excitations mostly remains, though some exceptions appear, and a few excited states show excitations delocalized between the two sides of the dimer. This comes from the well-known fact that the geometries of the Z isomers are not perfectly planar so that the spatial orthogonality of both moieties is strictly no longer present beyond the EE isomer. As these states imply charge transfer between both moieties (H → F or F → H), they have very small transition probabilities, so their impact in the photochemical behavior of these molecules will be negligible. An additional effect of the loss of planarity of the Z isomers is that the classification of the electronic excitations as nπ* or ππ* is not possible beyond the EE isomer. However, it is clear that for all the isomers two bands are clearly observed: A very intense one at high energies that loosely corresponds to the expected ππ* allowed transition in azobenzene and a much less intense band that could be classified as nπ*. The shapes of all the molecular orbitals implied in the electronic excitations of the other isomers are depicted in Figures S1–S3.

The remarkably good agreement of the experimental absorbance electronic spectra of the E/Z isomers of dimer 1 with our calculations gives us confidence to tackle the much more complex study of the actual photochemistry of azobenzene multi-state photoswitches when irradiated by light of different wavelengths. To approach this goal, additional calculations have been performed for dimer 1. First of all, the transitions states for the different isomerization processes in the ground state have been exactly located. A second type of calculation, relevant to study the photochemistry of dimer 1, is the relaxation of the geometries in the excited electronic states.

**Figure 3:**
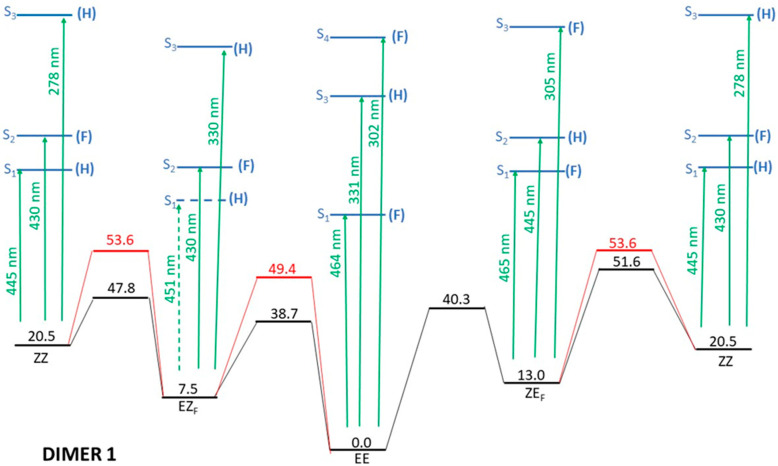
Full energy scheme of the ground and lowest excited electronic states of dimer 1. See text for details. Relative energies of the different structures (horizontal lines) are given in kcal/mol. Green arrows indicate the wavelengths of the vertical excitations from each conformer. Black lines indicate the ground electronic state whereas excited electronic states are in blue. Red lines correspond to crossing sections between S_0_ and S_1_.

Figure 3 contains all relevant data computed so far so that the discussion of what happens upon excitation of the more stable EE isomer (the only one present previous to any light irradiation) with light of different wavelengths can be undertaken. Again, the goal is to understand the reason behind the observed experimental facts so that we can use our theoretical results to design brand new azobenzene-based multiple photoswitches with improved properties.

Figure 3 is quite complex as it includes a large number of relevant data, so prior to any discussion, it is important to explain what is to be seen (and what is not to be seen!) in the figure. Horizontal lines indicate different states of energy (not necessarily energy minima as it is explained below). The energy of each structure is indicated in kcal/mol. Energies are relative to the most stable structure: The EE isomer in the ground electronic state. Energies of the four optimized isomers in the ground state are depicted in black. The transition state for the interconversion between two of these isomers is also depicted in black and connected through sloped straight lines to the corresponding minima. As the ZZ isomer can be obtained either from the EZ_F_ or the ZE_F_ forms, it appears twice at both the right and left edges of the figure, though it is obviously the same structure. Above each E/Z isomer the energies of the vertical transitions with low energy (basically the ones already analyzed in Table 1) and non-zero oscillator strengths are indicated by blue horizontal lines. For the EZ_F_ isomer, the transition to the lowest excited electronic state S_1_ is forbidden (f = 0.00), but this state is nevertheless shown as its presence may be relevant in the deactivation processes following irradiation. This fact is graphically displayed in Figure 3 by the use of a dashed blue line and a dashed green arrow indicating the impossibility of a direct excitation to this state. Each excited state has an energy value and a parenthesized letter (H or F) to indicate in which side of the dimer the electronic excitation is localized. The wavelengths of the vertical transitions are also indicated by green arrows. Finally, the lines and numbers in red indicate the approximate location of the conical intersections between the ground and the lowest singlet excited electronic states.

**Figure 4:**
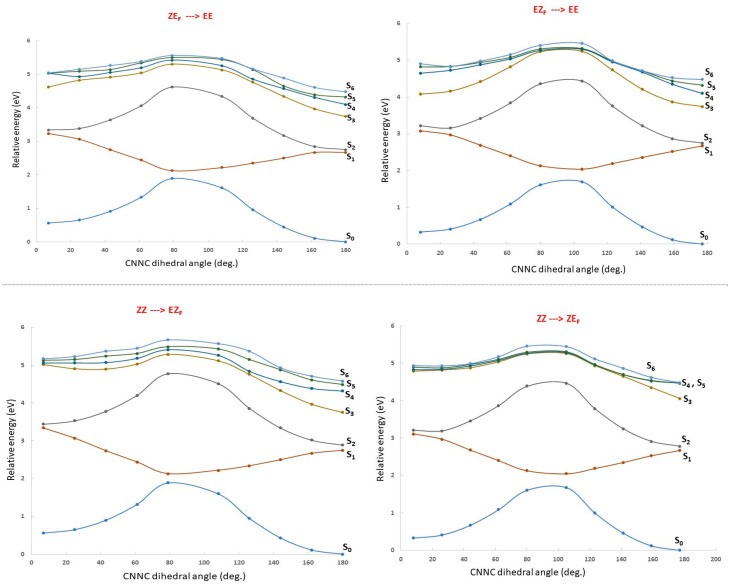
Relaxed scans of the potential energy surfaces of the ground electronic states along all the isomerization pathways (the CNNC dihedral angle of each azobenzene unit). At each point of the scan the energies of the lowest six excited electronic states are computed. The lines are drawn with different colors and adiabatically follow the energies of each electronic state so that they never cross.

To gain insight into the mechanism of the different isomerizations, relaxed scans were obtained by taking the CNNC dihedral angle of each azobenzene moiety as the reaction coordinate and optimizing the rest of the geometrical parameters in the ground electronic state. For each geometry obtained this way, a single-point calculation of the excited state was performed. Figure 4 displays these scans for the four available isomerization reactions. The electronic states up to S_6_ are followed adiabatically so the lowest energy corresponds to the ground electronic state S_0_, the second to S_1_ and so on. The states are specifically labeled in Figure 4. All the graphics show a clear decrease of the energy gap between S_0_ and S_1_ that becomes almost degenerate when the two phenyl rings are orthogonal (CNNC dihedral angle of 90°). Conversely, S_2_, that is quite close to S_1_ at both ends of the scan (close to the corresponding isomer minimum), maintains the energy gap with S_0_ all along the scan and this is also true for the higher excited states depicted in Figure 4.

The quasi degeneracy of S_0_ and S_1_ right in the middle of the reaction coordinate is a clear indication of the presence of conical intersections (CIs). CIs are effective funnels that promote the radiationless deactivation of the excited state to either side of the isomerization path and so their presence is relevant in order to grasp the photochemistry of any molecule. Approximate geometries of the CIs for dimer 1 were obtained through full optimization of the first excited electronic state near the crossing point. This usually led to points with almost null excitation energies where the optimization fails or enters a loop. Given the multiconfigurational character of these crossing regions, our theoretical procedure is not able to fully characterize these points, so the CI energies, depicted in red in Figure 4, and the geometries, shown on Figure S4, have to be taken with caution. For the sake of our discussion on the global mechanism of the multi-state photoswitches, the presence of such low-lying CIs is relevant as an indication of the presence of internal conversion, but a more precise and detailed analysis is not necessary.

In the experimental work of dimer 1, three different wavelengths (350 nm, 410 nm and >500 nm) are used to irradiate the EE isomer. This triggers some interconversions, but some remain absent [30]. In the following, we analyze the photochemical paths open at each irradiation in light of our theoretical computations. In the following discussion it is assumed that, as virtually all accessible excited states show an excitation localized in just one side of the dimer, it is that part of the dimer the one that is likely to isomerize upon photoexcitation to this state either through rotation/inversion in the excited state either by internal conversion through a conical intersection when present).

- Irradiation with λ > 500 nm: This low-energy radiation is slightly below the lowest calculated energy excitation of the EE form from S_0_ to S_1_ (464 nm), but taking into account the error bars of the calculations and the broadness of the electronic transition bands, we can assume that this radiation allows the excitation of the EE initial form to S_1_. The oscillator strength of this transition is quite small (0.1), but such a value is usually considered enough to permit the electronic transition. Once in the S_1_ excited state, the molecule will lose energy and eventually return to the ground electronic state, emitting radiation to return to the original EE structure or else by internal conversion passing through the CI that links EE to EZ_F_. This would explain the predominance of the EZ_F_ structure as a result of this irradiation.
- Irradiation with λ = 410 nm: This radiation does not produce any noticeable isomerization reaction as the EE isomer is the dominant species after irradiation. This is easily explained from our results as there are no allowed excitations close to this value for the EE isomer.
- Irradiation with λ = 350 nm: In this case, it is experimentally found that the final majority product is the ZZ isomer. This seems anomalous as in order to obtain the ZZ isomer from the initial EE one, two consecutive isomerizations are needed: The first one from EE to either EZ_F_ or ZE_F_ and a second one from each one of these “crossed” isomers to ZZ. According to the results shown in Figure 3, this wavelength should clearly excite the largely allowed transition of EE to S_3_ and probably also the transition to S_4_, again with a large f value even if it is slightly above in energy. From our reasoning, this would mean that both EZ_F_ and ZE_F_ isomers could be obtained. To explain the formation of the ZZ isomer, a second radiative process should sequentially occur. Our results allow for this to occur given that the 350 nm irradiation can photo-excite both the EZ_F_ and ZE_F_ isomers as the two crossed isomers have mainly allowed transition at values slightly above 300 nm. In both cases this second electronic absorption would allow for the isomerization leading to the ZZ isomer. Previous literature extensively reports the feasibility of using such a stepwise, two-photon absorption process to fuel specific photochemical processes of interest [35,36,37]. Parenthetically, we note that at λ > 500 nm, the ZZ adduct is also experimentally obtained as a secondary but noticeable adduct (18%). Its presence should also be explained as the product of two consecutive irradiations of the same energy.

In addition to the photoexcitations allowed for the EE isomer, there are additional transitions between pairs of isomers that are also possible according to the experimental results:

- EZ_F_ → EE and ZE_F_ → EE at λ = 410 nm: At this wavelength EZ_F_ could access the S_2_ state. Given that this state implies excitation of the F part it could isomerize to EE. Almost the same pattern can be invoked to explain the isomerization of ZE_F_ back to EE.
- EZ_F_ → ZZ and ZE_F_ → ZZ at λ = 350 nm: These two transitions have been already discussed as the second step of the sequential two-photon absorption from EE to ZZ. The high energy irradiation would allow the excitation of the most probable transition (highest value of f) for both crossed structures where isomerization to the ZZ isomer is feasible.
- ZZ → EE at λ = 410 nm: We encounter again a double isomerization process so that, according to our scheme, we will have to claim for a sequential double photo-absorption. For the ZZ isomer the transition to S_2_ is calculated at 430 nm and at this state the ZE_F_ forms could be obtained. A quite close wavelength of 445 nm would excite the ZE_F_ also to S_2_, where the isomer EE could be finally obtained. The same process could be envisaged to take place also in the inverted order: ZZ → EZ_F_ → EE, implying successive light excitations of 445 and 430 nm (that is: identical values of the former process but also inverted!).
- ZE_F_ → EZ_F_ at λ > 500 nm: This seems the most difficult transition to explain as it has to involve a sequential double-electronic excitation by means of two low-energy photons. However, our calculations nicely explain this process assuming that transitions calculated at wavelengths up to 440 nm can be accessed by the considered irradiation. In fact, as in the previous case, we have two possible paths and both are feasible: ZE_F_ → ZZ → EZ_F_ with absorptions predicted at 465 and 445 nm, respectively, and ZE_F_ → EE → EZ_F_ with two consecutive absorptions now calculated at 445 and 464 nm (curiously enough, again almost the same values but inverted!).

In addition to the photochemistry of dimer 1 just discussed, we may also further analyze the isomerization mechanism in the ground electronic state. As seen in Figure 3, the energy barriers for the isomerizations in the ground electronic state are high enough (larger than 38 kcal/mol) to guarantee the relatively large stability of the final isomer that, eventually, will thermally revert to the most stable EE structure. In fact, it is found experimentally that half-lives of the unstable isomers obtained after light irradiation range from days to years [30]. This conclusion is not modified if we take into account the thermodynamic corrections. Table S1 of the Supplementary Material gives the enthalpies and Gibbs free energies of the four isomers and the corresponding transition states (using the quite drastic rigid rotor and the harmonic oscillator approximations). The nature of the transition states can also be disclosed by analyzing the direction of the transition vector (the motion that corresponds to the only one imaginary frequency in the localized transition states). In all the cases the motion of this imaginary frequency corresponds to an in-plane inversion of the N=N-C angle with the three atoms almost aligned in the transition state structure. Then, the inversion mechanism prevails over the torsion (rotation of the full phenyl ring) as already observed in previous systematic theoretical studies of different azobenzenes [38].

From the comprehensive analysis of dimer 1 just performed, we have obtained a profound insight of the physical reasons behind the quite complex photochemical behavior of this molecule. This analysis can now be used to our main goal: The design of more efficient azobenzene-based multi-state photoswitches whose properties could be modified “à-la-carte” using a computationally guided protocol. It is not easy to manage all factors affecting the photochemistry of a given multi-state photoswitch. A factor that appears as primordial, in order to be able to selectively excite the two AB moieties, is to pick up two monomeric units with absorption bands as separated as possible. Also, foreseeing the use of the designed photoswitch to be used in life sciences, the excitation sources should be kept at the lowest possible energy range. To selectively interconvert these structures by means of light irradiation is not an easy task as the absorption bands are usually quite broad, so a large energy separation between each pair of absorption bands is compulsory. Besides, to simultaneously isomerize both units, a double photon-absorption must take place. If the corresponding absorption bands are close enough, a single irradiation could accomplish such a goal (such a mechanism has been invoked previously to explain the behavior of dimer 1), but this contradicts the otherwise necessary large separation of bands to selectively isomerize each side of the dimer. Here, we open the door to a possibility, not considered from the experimental side: The irradiation of the sample with two different wavelengths that would trigger the two consecutive E/Z isomerizations.

**Scheme 2:**
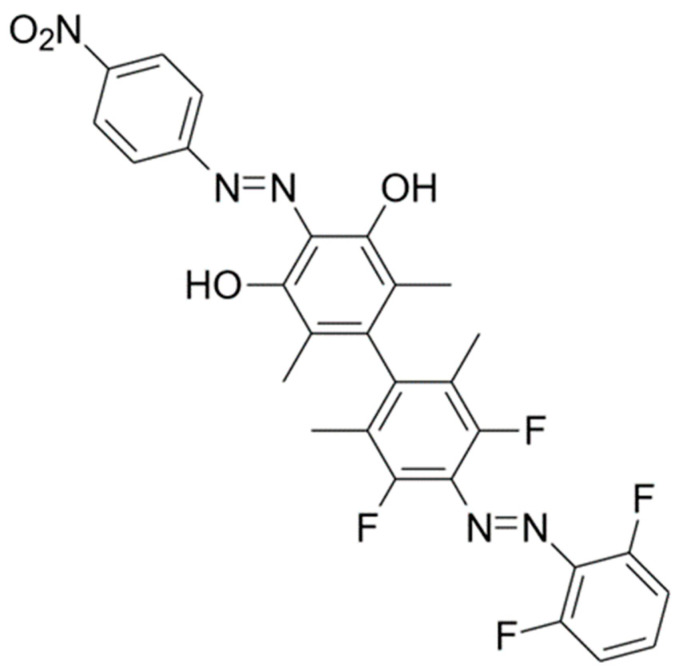
Molecular structure of dimer 2 in its most stable (EE) form.

Let us now use these generic ideas to conceptually design an azobenzene multi-state photoswitch where all the isomerization paths could be selectively photoactivated. The experimental and theoretical analysis of dimer 1 has clearly shown that the absorption spectra and isomerization ratios of the dimer are very similar to the sum of the photochemical properties of the two isolated azobenzenic monomers [30], so in order to design new molecules, we can rely on the well-known behavior of substituted single azobenzene systems and the expected effect of different substituents. It is well known that azobenzene functionalized with four ortho-fluorine atoms results in a larger separation of the absorption bands of the E and Z isomers, so we keep that AB unit in our newly designed four-state photoswitch. As for the other moiety, we introduce a strong electronic-acceptor NO_2_ group in para and two electron-donor hydroxyl groups in ortho position in the other phenyl ring. This design is typical of a push-pull molecule. It is well known that the presence of such antagonistic substituents on opposite sides of the molecule dramatically shortens the HOMO–LUMO gap, so that the bands in the absorption spectra could be considerably red-shifted [39]. Previous studies of single azobenzenes with push-pull substituents have confirmed this well-established effect [40,41]. Finally, all the ortho positions separating the two ABs have been substituted with methyl groups to reinforce the orthogonality of the two moieties. The dimer so designed, called dimer 2 from now on, is depicted in Scheme 2.

First of all, we proceeded to perform a study similar to the one undertaken for dimer 1. As dimer 2 has not been yet synthesized, there is no experimental data to compare with and Table 2 gives all the relevant information of the calculated energies and characteristics of the excited state so that direct comparison with the same calculations for dimer 1 (presented in Table 1) can be done. The two moieties of the dimer are now identified where necessary with subindex F (ortho-fluorinated part) or P (push-pull part).

**Figure 5:**
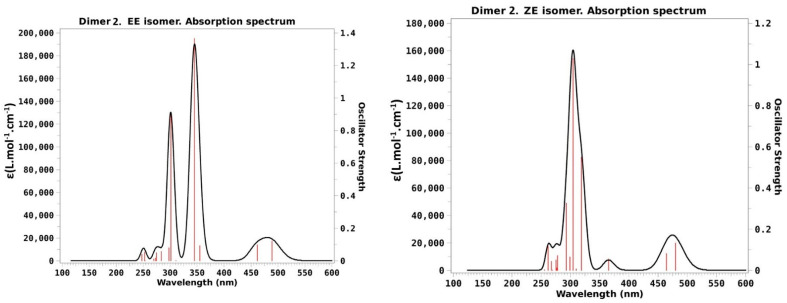

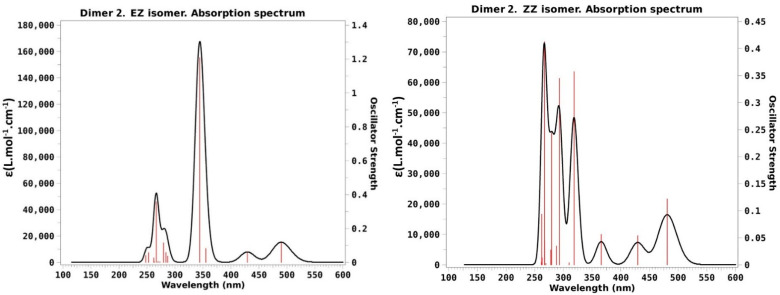
Computed absorption spectra of dimer 2 in acetonitrile at the optimized ground-state geometries of the four stable isomers of dimer 2. Vertical red lines indicate the position of the vertical transitions while the black contour corresponds to the convolution with a Lorentzian function depicted in order to theoretically reproduce the experimental absorption spectrum.

As done for dimer 1, we can use the calculated values posted in Table 2 to draw theoretical absorption spectra of dimer 2. The results are presented in Figure 5 for the four stable isomers of dimer 2.

**Figure 6:**
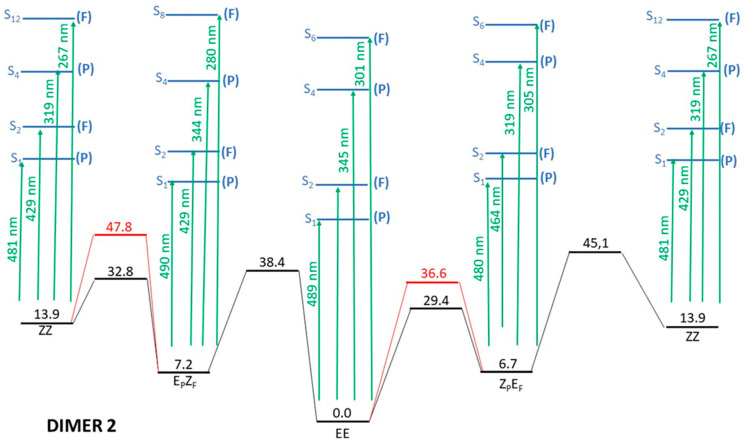
Full energy scheme of the ground and lowest excited electronic states of dimer 2. See text for details. Relative energies of the different structures (horizontal lines) are given in kcal/mol. Green arrows indicate the wavelengths of the vertical excitations from each conformer. Black lines indicate the ground electronic state whereas excited electronic states are in blue. Red lines correspond to crossing sections between S_0_ and S_1_.

To proceed to the more relevant point here (the expected photochemical behavior of the newly designed multi-state photoswitch), we have to turn the attention to Figure 6. It is identical to Figure 3 but with the data of dimer 2 (as presented in Table 2). This figure contains all the relevant information for the analysis of the photochemistry of dimer 2, which will be used from now on.

A simple and quick inspection of Figure 6 and its comparison with the analogous Figure 3 for dimer 1 reveals that the first goal of our design is fulfilled, as now for each isomeric form there are four transitions with more or less relevant (f ≥ 0.1) oscillator strengths that correspond to the selective excitations of just one unit of the dimer, either through what can be loosely classified as an nπ* excitation (low energies but also low probabilities) or through ππ* excitations (higher energy but also much higher probability). Additionally, we note that the isomerization barriers in the ground electronic state remain high enough (though they are slightly lower compared to the ones for dimer 1 shown in Figure 3) to ensure long enough stability of the metastable isomeric structures which, in turn, are slightly more stable as compared with dimer 1. As for dimer 1, the thermodynamics of the ground electronic state does not qualitatively modify the energetic scheme of dimer 2 seen in Figure 6 (see Table S2). The analysis of the transition vectors in the four located transition states also confirms the inversion mechanism for the isomerization reactions in the ground electronic state.

**Figure 7:**
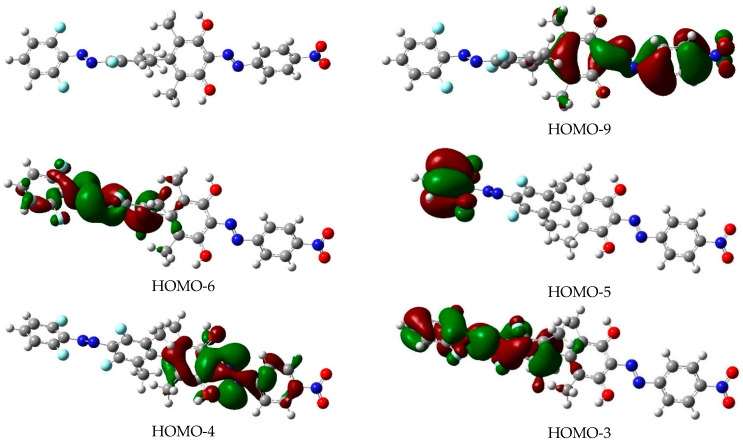

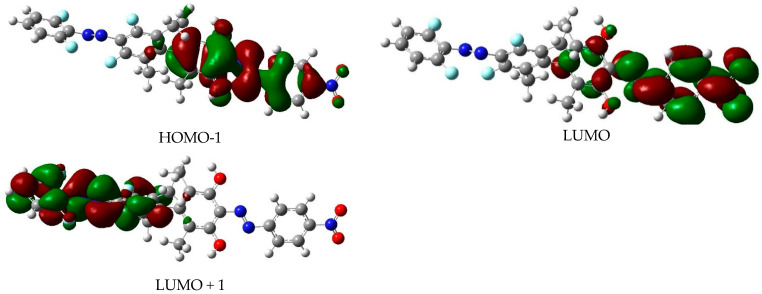
Shape of the molecular orbitals involved in the relevant excited electronic states of the EE isomer of dimer 2. The drawings correspond to an iso-level of 0.02 a.u. of electronic density. The left top corner of the Figure depicts the molecular structure of the EE isomer.

Depictions of the molecular orbitals involved in the lowest-energy relevant excited electronic states of the EE isomer are shown in Figure 7. As for dimer 1, the shape of the orbitals allows for an easy assignation to either being in the corresponding benzenic plane (n) or else located symmetrically above and below this plane (π). They are also clearly localized in just one moiety of the dimer so that orthogonality of the electronic excitations (as seen in dimer 1) is preserved in the proposed new dimer. The corresponding orbitals for the other three isomers of dimer 2 are to be seen in Figures S5–S7. As done for dimer 1, the approximate geometries of the localized conical intersections (red horizontal lines in Figure 6) are presented in Figure S8.

**Figure 8:**
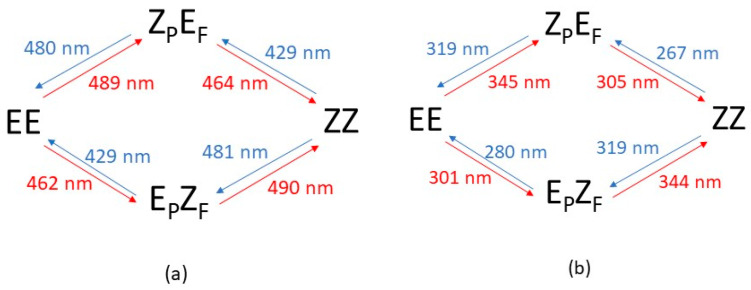
Allowed photo-isomerizations for dimer 2. (**a**): Activated through low-energy photoexcitation (λ > 425 nm). (**b**): Activated through high-energy photo-excitation (λ < 350 nm).

As we do not have any experimental data to compare with, it is not necessary to specifically analyze each feasible isomerization reaction. A more efficient way to grasp which processes can be photoactivated and which irradiation will activate them is the scheme depicted in Figure 8, which shows all the possible single photon reactions and the wavelength that is predicted to trigger each one of them. Again, we will assume that the azobenzene part that is photoexcited is the one that is able to eventually isomerize. As now there are always two such photoexcitations, involving either the nπ* low-energy or the ππ* high-energy electronic excited states, Figure 8 is divided into two parts: Part (a) on the left side corresponds to low energy excitations (λ > 425 nm) whereas part (b) on the right depicts the processes activated through high energy excitations (λ < 350 nm).

A naïf interpretation of the data seen in Figure 8 would conclude that for dimer 2 one should be able to selectively trigger any isomerization process involving just one monomeric part as, strictly speaking, each process has a different activation wavelength. However, this conclusion overlooks that the actual absorption bands are known to be quite broad (as previously noted when dealing with dimer 1). The problem is that the actual extent of a given electronic band is difficult to assess from a purely theoretical point of view as it would be necessary to proceed to a careful (and quite time-consuming in terms of computer time) analysis of the vibrational structure of the electronic bands, and the values given in Figure 8 (and elsewhere in the paper) just provide the calculated position of the maximum of the electronic absorption band. In addition to that, solvent and external conditions are also going to enlarge the broadening to an extent that is even harder to evaluate from a purely theoretical view. At any rate, it is obvious that where two absorbing bands have a more separated maxima it is more likely that they could be selectively excited. Comparison of the allowed excitations between the original dimer 1 and dimer 2 proposed here (Figure 3 and Figure 6) clearly discloses that in dimer 2 the calculated excitation wavelengths of the two moieties of the dimer are more separated in both the high (ππ*) and low (nπ*) energy regimes. Additionally, in dimer 2 all the isomeric species have four allowed excitation bands (with oscillator strengths greater than 0.1), whereas in dimer 1 just three of them (two for the EZ_F_ isomer) are allowed. This difference is in favor of dimer 2 as it opens the door for more photochemical paths to excite a particular isomer.

Within the limitations just explained, we can now proceed to a specific analysis of the open ways to photoexcite each isomeric species of dimer 2. In the following discussion it will be assumed that an energy separation between two absorption bands larger than 0.5 eV is high enough to permit selective excitation, whereas differences lower than 0.2 eV imply that both moieties of the dimer will be jointly photoexcited. As for intermediate energy separations, the possible selective excitation, as discussed before, will depend on the unknown shape of each absorption band.

- **Irradiation of the EE isomer:**

As this is the most stable and, presumably, only present form for dimer 2 in the absence of irradiation, the most relevant photochemical processes are taking place departing from this structure. At the long wavelength side of the spectrum (Figure 8a), it is seen that there are two allowed processes at 489 and 462 nm that should mainly lead to Z_P_E_F_ and E_P_Z_F_ isomers, respectively. The 0.14 eV difference between both radiations is not enough to allow a selective excitation of both isomers. As for the EE → ZZ process it will need a sequential process involving an initial photo-excitation to either Z_P_E_F_ or E_P_Z_F_ forms that would be, almost immediately, excited by a second photon. These processes are predicted to involve two absorptions of 489 and 464 nm for the ZZ isomerization through Z_P_E_F_ or 462, and 490 nm if it goes through E_P_Z_F_. In both cases, the two irradiations are quite close (0.14/0.15 eV) and it is likely that experimentally their bands will overlap so that a single irradiation near the 500 nm mark (the one used to photoexcite dimer 1) may allow for the double EE to ZZ isomerization process.

Things are different for the high-energy irradiation of EE (Figure 8b). In that case, irradiations of 345 and 301 nm will mainly lead to Z_P_E_F_ and E_P_Z_F_, respectively. The noticeable energy difference of these two bands (0.53 eV) now seems enough to guarantee selective excitation of the EE initial structure. As for the double isomerization to ZZ, it would need two photon absorptions either at 345 and 305 nm (if passing through Z_P_E_F_) or 301 and 344 nm (passing through E_P_Z_F_). Both paths have now quite similar energy differences between the two predicted absorption bands of ca. 0.5 eV, a value also high enough to anticipate that, within our theoretical results, the process may not proceed with just one irradiation wavelength. At this point, an alternative procedure, not considered in the experimental setup used for dimer 1, is envisaged: Irradiation of the sample with two different wavelengths that would trigger the consecutive two E/Z isomerizations.

- **Irradiation of the Z_P_E_F_ isomer:**

At the higher wavelength range, the calculations predict that this isomer could be irradiated by close wavelengths of 480 and 464 nm to obtain the EE and ZZ structures, respectively, so it may be impossible to selectively obtain just one form with low-energy irradiation. To directly produce the E_P_Z_F_ crossed isomer, two sequential absorptions of 480 + 462 nm or 464 + 481 nm are needed. The latter implies just a 0.09 eV difference between both bands, so it should be possible to accomplish the two consecutive photoexcitations with a single wavelength again near the 500 nm mark.

As for the high-energy range of photoexcitations, Z_P_E_F_ isomer may be photoexcited at 319 and 305 nm, respectively leading to EE and ZZ isomers. Again, the two radiations appear to be too close in energy to guarantee selectivity in the isomerization through irradiation. As for the two possible sequential two-photon processes necessary to obtain the E_P_Z_F_ isomer, they are predicted at sequences of 319 + 301 nm or else 305 + 319 nm. In both cases, the energy separation between both excitations (0.18/0.23 eV) does not clarify whether the double isomerization could be feasible with just one wavelength or if, depending on the experimental setup, two different irradiation frequencies might be necessary.

- **Irradiation of the E_P_Z_F_ isomer:**

In the long wavelength range of photoexcitations, this isomer can proceed to EE at 429 nm and to ZZ at 490 nm. As for the double photo-absorption leading to the Z_P_E_F_ isomer, the maxima of the two involved absorption bands are predicted either at 429 and 489 nm or 490 and 429 nm. The energy difference (ca. 0.35 eV) between these two bands does not allow us to assess if selective photoexcitation will be possible within this wavelength range.

Clear differences are seen in the high-energy range as E_P_Z_F_ could revert to EE and ZZ in a single process with irradiations of 280 or 344 nm, respectively, so now a selective photoisomerization should be possible (0.83 eV difference). As for the two absorptions needed to obtain the Z_P_E_F_, they would involve quite separate successive irradiations of 280 and 345 nm or else 344 and 267 nm, making the double isomerization process accessible only through a stepwise two-photon absorption process involving two different light sources.

- **Irradiation of the ZZ isomer:**

According to our calculations, the two single photoisomerizations from ZZ could be selectively addressed in the high-energy region as the wavelengths predicted to photoexcite ZZ to E_P_Z_F_ and Z_P_E_F_ are, respectively, 319 and 267 nm (0.75 eV away). Things are not so clear at the low energy range, where photoexcitation at 481 nm is predicted to lead to E_P_Z_F_, whereas irradiation at 429 nm (0.31 eV difference) will produce Z_P_E_F_.

As for the photochemical paths leading to the double isomerization from ZZ to EE in the low-energy domain, they are predicted at 429 and 480 nm or else 481 and 429 nm, an intermediate separation of around 0.31 eV in both cases that makes it again difficult to predict whether a single irradiation may trigger the process or if two different irradiations will be needed. Things are clearer at the high-energy range as the double isomerization implies well separated (>0.5 eV) sequential absorptions of 267 and 319 nm or else 319 and 280 nm.

## 3. Materials and Methods

All theoretical calculations have been done with the GAUSSIAN09 program [42]. Acetonitrile, the solvent used in the experimental work by Zhao et al. [30] is introduced in all the calculations performed through the use of the continuum PCM method as implemented in the GAUSSIAN09 suite of programs [43,44]. The geometries of all the stable isomers in the ground electronic states have been obtained through full geometry optimization at the DFT level within the hybrid long-range corrected CAM-B3LYP functional, which avoids the problems of non-long-range-corrected functionals (such as B3LYP) that grossly underestimate the excitation energies of excited states of internal charge-transfer type [45,46]. In the recent past, CAM-B3LYP has been proved to perform well for organic molecules of moderate size with a systematic tendency to overestimate excitation energies [47,48,49]. This error has the attractive feature of being quite constant [47]. Recent benchmarks analyzing a large set of molecules confirm CAM-B3LYP among the best functionals to accurately measure vertical excitation energies [50,51]. Additional technical details of the calculations may be found in the Supplementary Material.

Frequency calculations have been carried out at the optimized geometries in the ground electronic state to confirm that they are true minima (all eigenvalues of the second derivative Hessian matrix are positive). Transition states for the isomerization reaction in the ground state have also been directly located and fully characterized (with exactly one negative eigenvalue of the Hessian matrix).

Electronic excited states have been obtained using the linear time-dependent formalism at the DFT level (TD-DFT) without the Tamm–Dancoff approximation. The basis set used is 6-311+G(d,p), a triple-ζ quality split-valence that also includes polarization and diffuse functions [52,53]. We have tested that the use of the larger def2-TZVP basis set [54,55] does not appreciably modify the geometries and the relative energies of the different isomers and the same holds for the calculated excitation energies as differences between the two basis sets well below 0.1 eV, as posted in Tables S3 and S4 in the Supplementary Material. Geometry optimizations in the excited state were unsuccessful as the optimization systematically turned to points with almost null excitation energies where the optimization failed or entered a loop. This is a clear indication that after photoexcitation the geometry relaxes to a point where it crosses the potential energy surface of the ground electronic state. These points are called conical intersections (CIs) and their presence is known to be ubiquitous in almost every photochemical process involving E/Z isomerizations [56,57,58]. A more precise localization of the CI structures is not possible within our single-reference DFT method as it would require a multi-determinantal methodology, but, as discussed in the results and discussion section, this precision is also not necessary in the present context.

Cartesian coordinates for all the located geometries of interest (minima, transition states and conical intersections for both dimer 1 and dimer 2) are given in Tables S5–S25.

## 4. Conclusions

In this work, we have performed high-level computations devoted to rationalizing the photochemistry of multi-state photoswitches. This task has allowed the design of a conceptual and computational mechanism to propose brand new molecular architectures with specific properties. To do so, we have first considered a bisazobenzene system (dimer 1), well-studied from the experimental side. We have carefully analyzed from a theoretical point of view the relevant features of this molecule. Comparison of the obtained data with the experimentally known facts has allowed us to gain full comprehension of the mechanisms governing the photochemistry of this four-state photoswitch.

The main target of such a multi-state photoswitch is that the different isomers could be selectively interconverted using different sets of light irradiations. In dimer 1 this goal was just partially accomplished as only two out of three isomers were obtained from the most stable isomer with irradiation of different wavelengths. Moreover, some other interconversions between isomers were not experimentally observed to occur at any of the considered irradiations. The deeper insight acquired from our computational design has allowed us to attribute these drawbacks to the fact that the energies of the photons needed to excite each azobenzenic unit are not separated enough to allow selective isomerization. Combining this strategy with the huge wealth of knowledge about the influence of different substituents on the electronic excitation energies of single azobenzenes, a new bisazobenzene (dimer 2) that should overcome the shortcomings of the previous one is proposed.

Calculations on dimer 2 confirm the expectations (to be experimentally confirmed) of our computationally guided design. Computations predict that each isomer may absorb light of four different wavelengths: Two of high energy (λ < 350 nm) and two of low energy (λ > 425 nm), in each case leading to excitations of a different azobenzene unit. This opens the door to more photochemical paths that may selectively address a given isomerization process. For dimer 1, just one excitation was allowed (f ≥ 0.1) in the low energy region for all the isomeric species. Departing from the EE isomer, the only thermodynamically stable species of dimer 2, our calculations predict that the initial, and more stable, EE structure could be selectively photoexcited to the other three isomers using light of three different wavelengths centered respectively at 301 nm, leading to E_P_Z_F_, 345 nm for the Z_P_E_F_ structure and 464 + 489 nm (probably just one single wavelength given the broadness of the absorption bands) to mainly obtain the ZZ isomer.

The fact that for dimer 2 the separation of the absorption bands of both moieties has been enlarged makes the absorption of two consecutive photons of the same energy, needed to isomerize both moieties of the dimer with a single irradiation, a process that in dimer 1 was the key to obtain the ZZ isomer from the initial EE one in the previous experimental work, less likely. The result obtained for dimer 2 has led us to propose a brand-new procedure to selectively access the different isomers of a multiazobenzene system not considered up to now: A simultaneous irradiation with two different light sources. Given that there are four isomeric structures, and so twelve possible interconversions between each pair of metastable structures, finding an ideal molecular system where each transition could be triggered through single irradiations of light with separated-enough wavelengths is an unattainable goal, so that the eventual use of pairs of excitations, as proposed here, may be a clever way to increase the available tools to selectively trigger such multi-step processes through different photoirradiations. Our computational strategy is able to deal with more complex systems with three (or more) “isomerizable” units. The star-like structure with three azobenzene units linked to a central core is a paradigmatic case that has aroused considerable interest in recent years [26,27,28,29]. A clever choice of different substituents at each branch should lead to a system with well-separated electronic absorption bands so that selective isomerizations could be accomplished using either one or two different irradiation wavelengths. Additional work towards this goal is in progress in our laboratory.

## Figures and Tables

**Table 1:** Relevant lowest electronic excited states of the four E/Z stable isomers of dimer 1.

Isomer	State	E (eV)	λ (nm)	f	Excitation
EE	S_1_	2.67	464	0.10	H−1 → L; H−7 → L (F)
	S_2_	2.75	451	0.00	H−2 → L+1 (H)
	S_3_	3.74	331	1.61	H → L+1 (H)
	S_4_	4.10	302	0.78	H−1 → L; H−7 → L (F)
EZ_F_	S_1_	2.75	451	0.00	H−2 → L+1 (H)
	S_2_	2.89	430	0.06	H−1 → L (F)
	S_3_	3.75	330	1.29	H → L+1 (H)
	S_4_	4.32	287	0.08	H−3 → L+1 (F → H)
ZE_F_	S_1_	2.67	465	0.09	H−1 → L; H−7 → L (F)
	S_2_	2.78	445	0.05	H → L+1 (F)
	S_3_	4.06	305	1.33	H−1 → L; H−7 → L (F)
	S_4_	4.47	277	0.07	H−4 → L; H−5 → L (H → F)
	S_5_	4.47	277	0.03	H−4 → L; H−5 → L (H → F)
	S_6_	4.48	276	0.16	H−2 → L+1 (H)
ZZ	S_1_	2.78	445	0.05	H → L+1 (H)
	S_2_	2.88	430	0.05	H−1 → L (F)
	S_3_	4.46	278	0.36	H−2 → L+1; H → L+1 (H)
	S_4_	4.50	275	0.02	H−8 → L (F)
	S_5_	4.57	271	0.12	H−4 → L (H → F)

**Table 2:** Relevant lowest electronic excited states of the four E/Z stable isomers of dimer 2.

Isomer	State	E (eV)	λ (nm)	f	Excitation
EE	S_1_	2.54	489	0.12	H−1 → L; H−4 → L (P)
	S_2_	2.69	462	0.10	H−2 → L+1; H−6 → L+1 (F)
	S_4_	3.59	345	1.36	H−1 → L; H−4 → L (P)
	S_6_	4.10	301	0.9	H−2 → L+1; H−6 → L+1 (F)
E_P_Z_F_	S_1_	2.53	490	0.11	H−1 → L; H−4 → L (P)
	S_2_	2.89	429	0.06	H−9 → L+1; H−2 → L+1 (F)
	S_4_	3.60	344	1.21	H−1 → L; H−4 → L (P)
	S_8_	4.44	280	0.11	H−5 → L+1; H−6 → L+1 (F)
Z_P_E_F_	S_1_	2.58	480	0.13	H−1 → L; H−1 → L+2 (P)
	S_2_	2.67	464	0.08	H−6 → L+1; H−2 → L+1 (F)
	S_4_	3.89	319	0.55	H−1 → L; H−5 → L+2 (P)
	S_6_	4.07	305	1.03	H−6 → L+1; H−2 → L+1 (F)
ZZ	S_1_	2.58	481	0.12	H−1 → L; H−1 → L+2 (P)
	S_2_	2.89	429	0.05	H−9 → L+1; H−2 → L+1 (F)
	S_3_	3.39	366	0.06	H → L; H → L+2 (F → P)
	S_4_	3.89	319	0.36	H−1 → L; H−5 → L+2 (P)
	S_12_	4.64	267	0.41	H−9 → L+1 (F)

## Data Availability

The data presented in this study are contained within the article.

## Supplementary Materials

The supporting information can be downloaded at: https:www.mdpi.com/article/10.3390/ijms23158690/s1.
