# Supplementary material for: On the Computational Design of Azobenzene-Based Multi-State Photoswitches
Source: Int J Mol Sci. 2022 Aug 4;23(15):8690. doi: 10.3390/ijms23158690 (PMC9369132; doi:10.3390/ijms23158690)
Supplement: Supplementary file 1 [file ijms-23-08690-s001.zip › ijms-1835196-supplementary.pdf]

On the computational design of azobenzene based multi-state photoswitches

Miquel Moreno [1,*], José M. Lluch [1,2] and Ricard Gelabert [1]

[1] Departament de Química, Universitat Autònoma de Barcelona, 08193 Bellaterra, Barcelona, Spain
[2] Institut de Biotecnologia i de Biomedicina (IBB), Universitat Autònoma de Barcelona, 08193 Bellaterra, Barcelona, Spain
* Correspondence: miquel.moreno@uab.cat; Tel.: +34935812174

SUPPLEMENTARY INFORMATION

Additional computational details ……………..……….……..…… 2

Shape of Molecular orbitals of dimer 1 ………………………..….. 3

Geometries of the conical intersections of dimer 1 ……………..... 6

Shape of Molecular orbitals of dimer 2 …………………..…….…. 7

Geometries of the conical intersections of dimer 2 ……………..… 10

Thermodynamics of the ground state for dimer 1 and 2 ……… 11

Results of dimer 1 using two different basis sets ………..…..... 12

Cartesian coordinates of the stationary points for dimer 1 ……. 13

Cartesian coordinates of the stationary points for dimer 2 …..... 29

Additional Computational details

The SCF convergence criteria for the calculations was stablished at 10^{-8} a.u. of energy. For the optimizations the convergence was granted when the larger gradient component was below $4.5 \cdot 10^{-3}$ a.u. and the RMS value was lower than $3.0 \cdot 10^{-3}$ a.u. For the DFT calculations a pruned grid with 99 radial shells and 510 angular points per shell was adopted. The linear TDDFT calculations were carried out without the Tamm-Dancoff approximation. The lowest 15 excited states were converged for each TDDFT run.

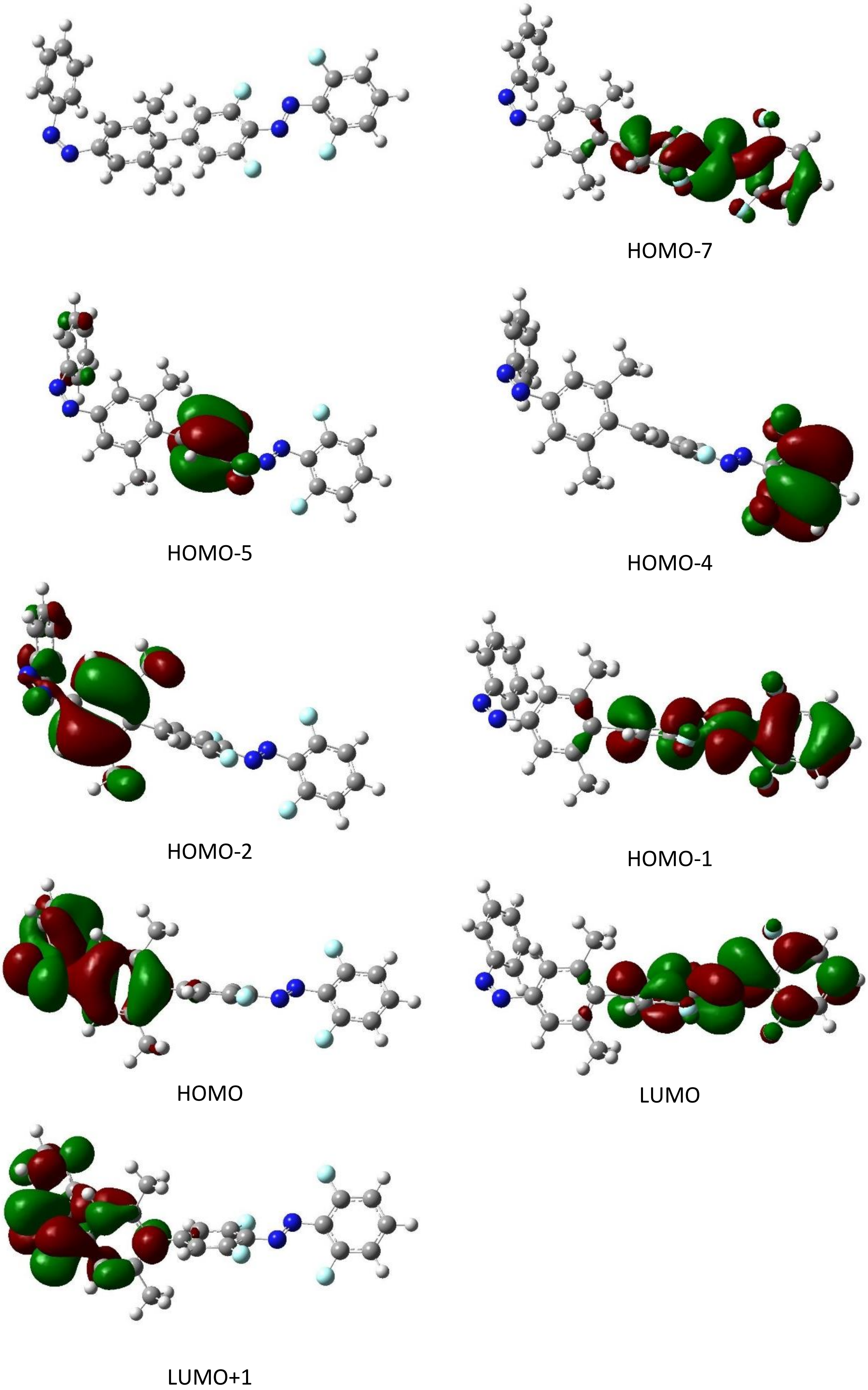

FIGURE S1: Shape of the molecular orbitals involved in the relevant excited electronic states of the ZEF isomer of dimer 1. The draws correspond to an iso-level of 0.02 a.u. of electronic density. The left top corner of the Figure depicts the molecular structure of the ZEF isomer.

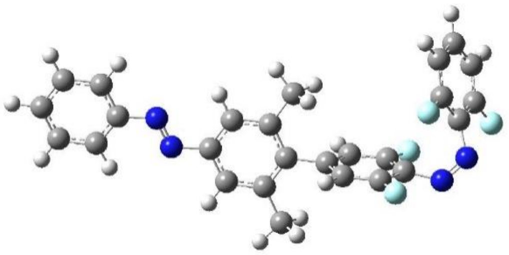

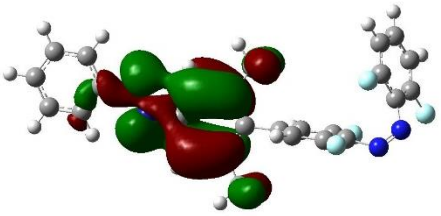

HOMO-3

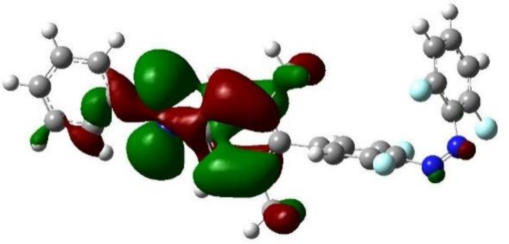

HOMO-2

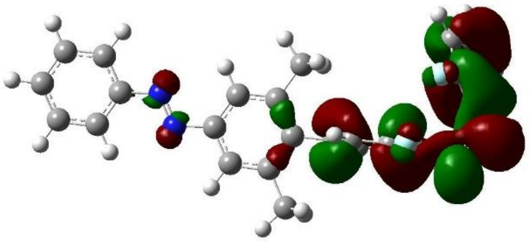

HOMO-1

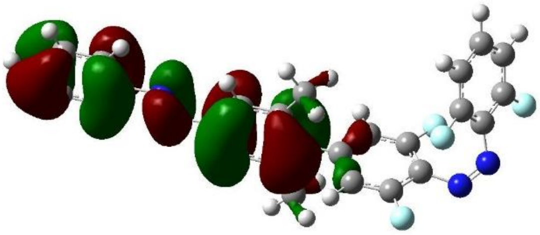

HOMO

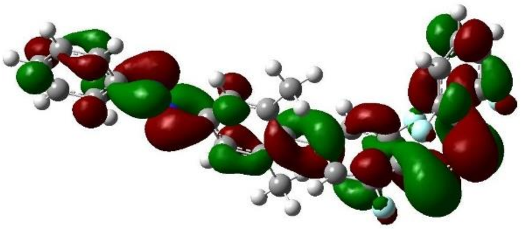

LUMO

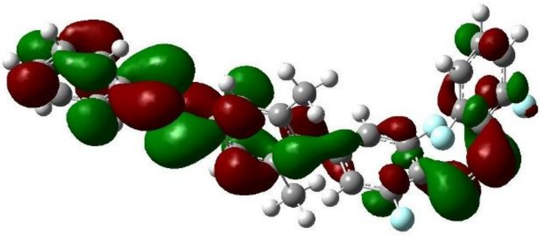

LUMO+1

FIGURE S2: Shape of the molecular orbitals involved in the relevant excited electronic states of the EZ_F isomer of dimer 1. The draws correspond to an iso-level of 0.02 a.u. of electronic density. The left top corner of the Figure depicts the molecular structure of the EZ_F isomer.

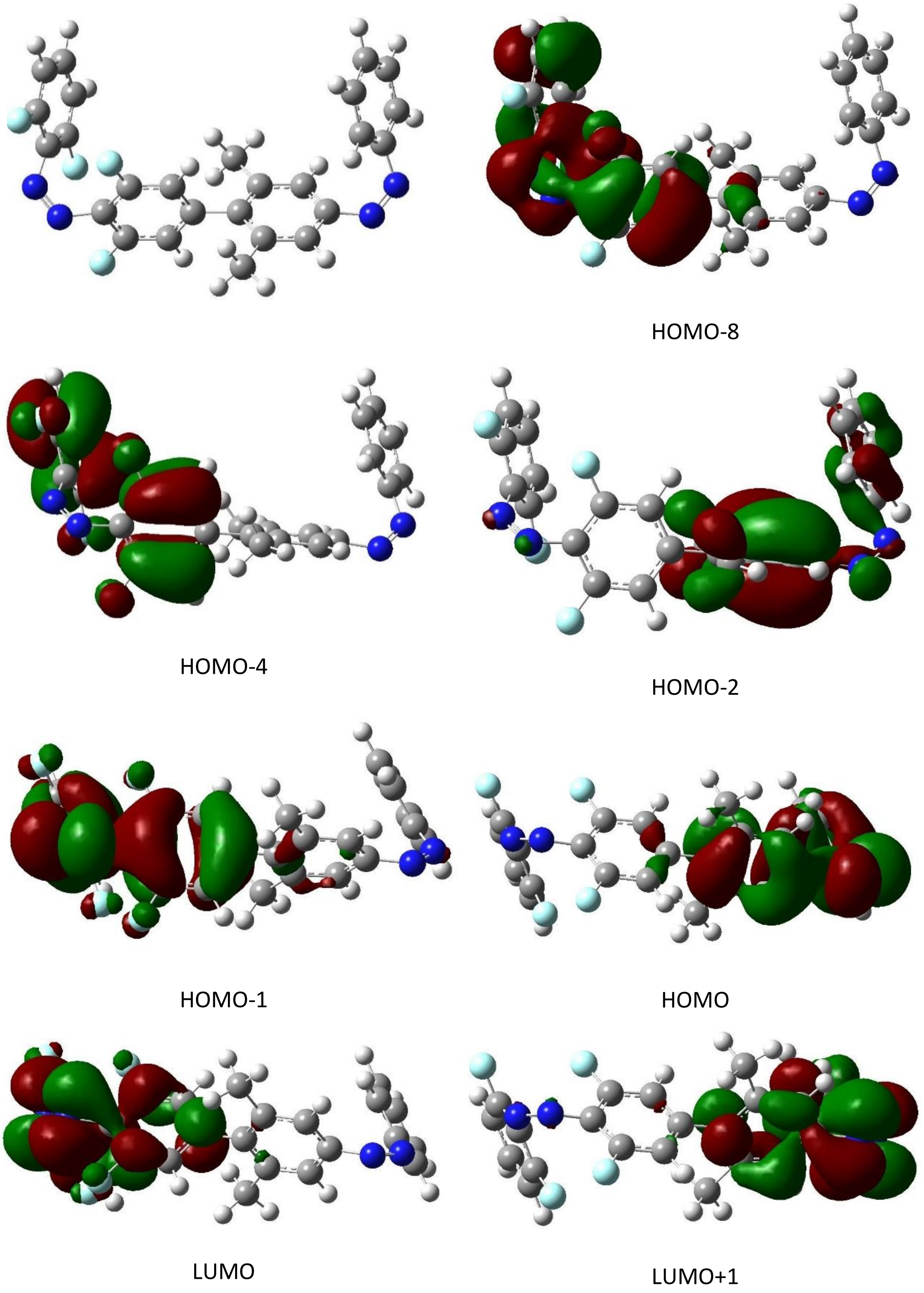

FIGURE S3: Shape of the molecular orbitals involved in the relevant excited electronic states of the ZZ isomer of dimer 1. The draws correspond to an iso-level of 0.02 a.u. of electronic density. The left top corner of the Figure depicts the molecular structure of the ZZ isomer.

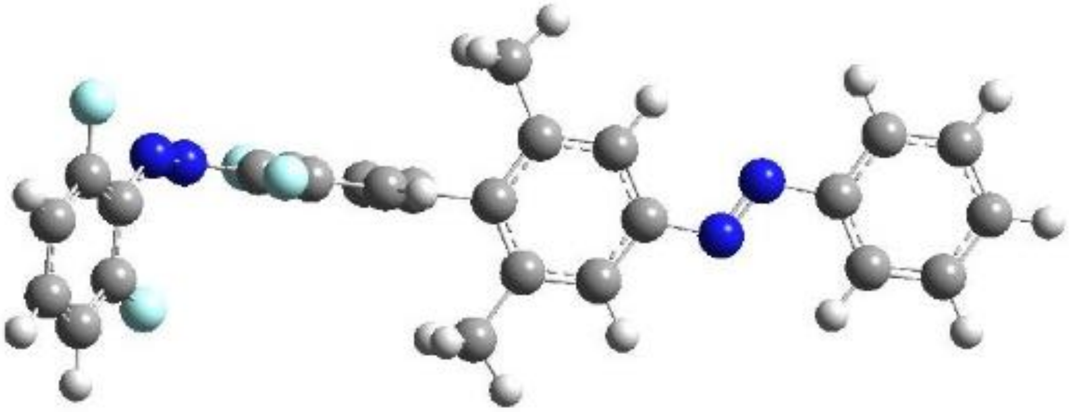

EE↔ EZ_F

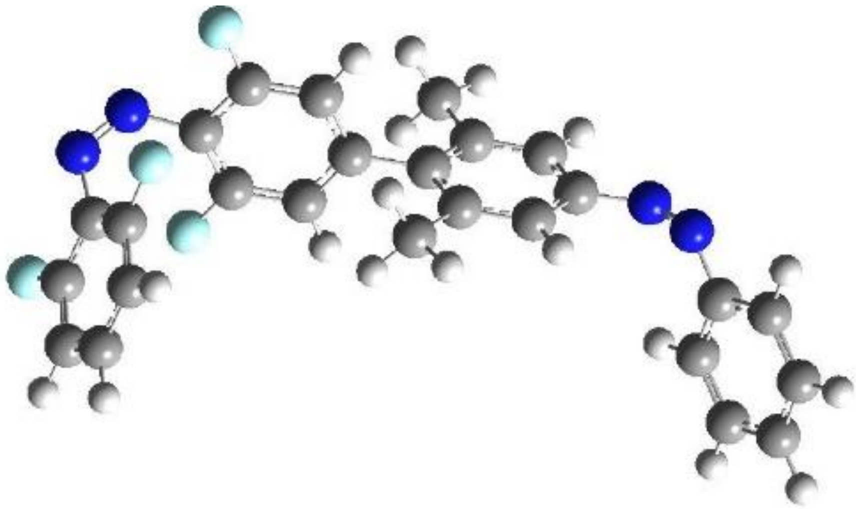

EZ_F ↔ ZZ

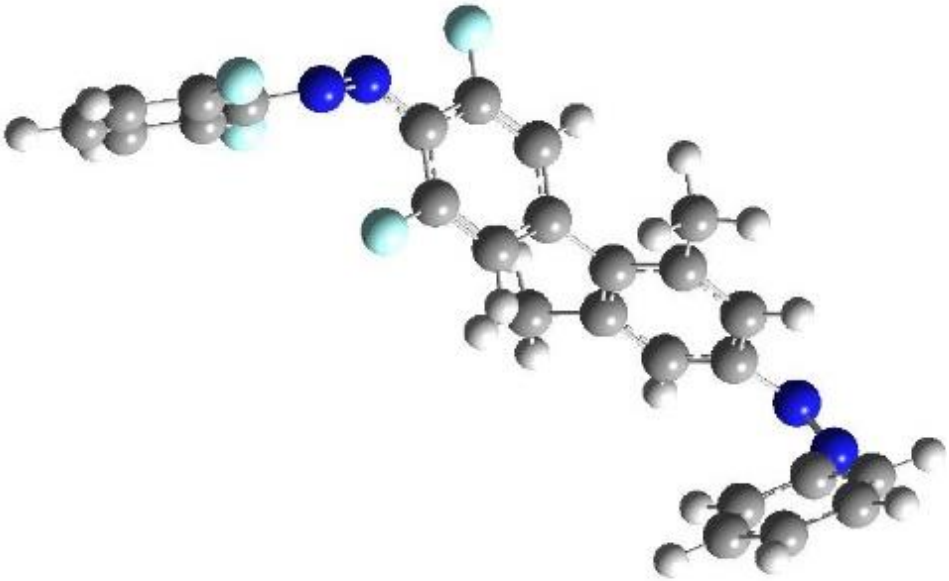

ZE_F↔ ZZ

FIGURE S4. Approximate geometries of the conical intersections localized for dimer 1.

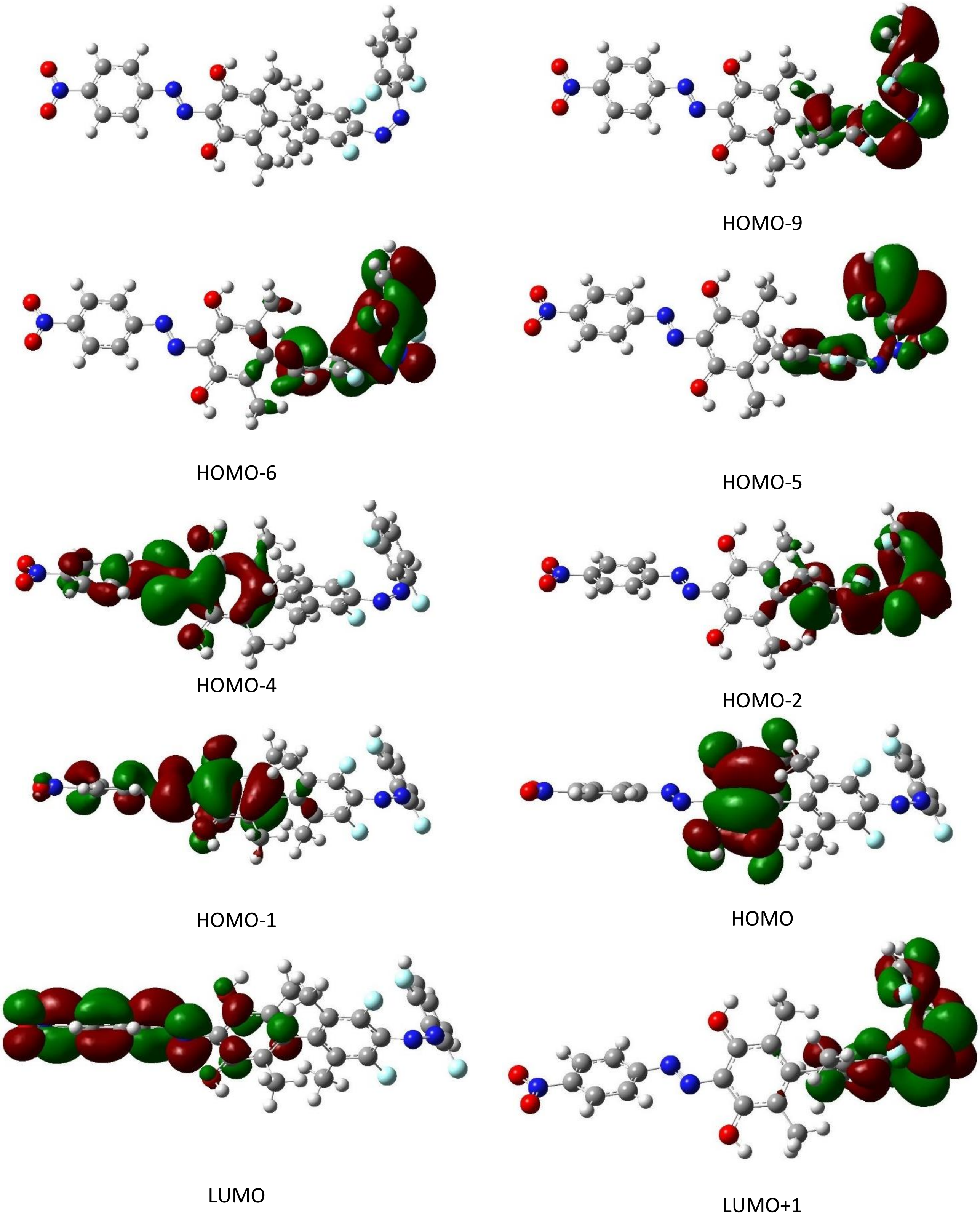

FIGURE S5: Shape of the molecular orbitals involved in the relevant excited electronic states of the E_PZ_F isomer of dimer 2. The draws correspond to an iso-level of 0.02 a.u. of electronic density. The left top corner of the Figure depicts the molecular structure of the E_PZ_F isomer.

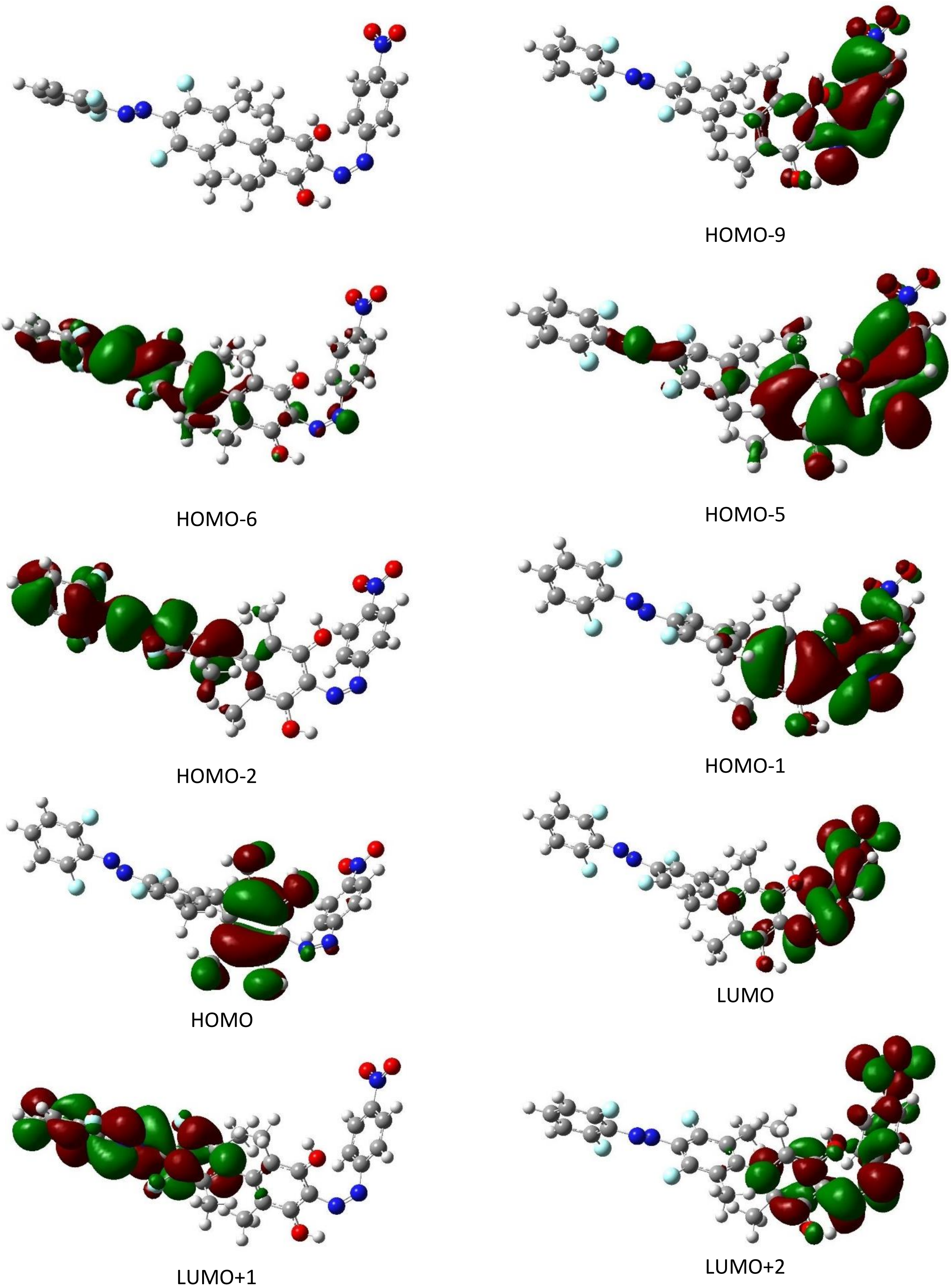

FIGURE S6: Shape of the molecular orbitals involved in the relevant excited electronic states of the Z_PE_F isomer of dimer 2. The draws correspond to an iso-level of 0.02 a.u. of electronic density. The left top corner of the Figure depicts the molecular structure of the Z_PE_F isomer.

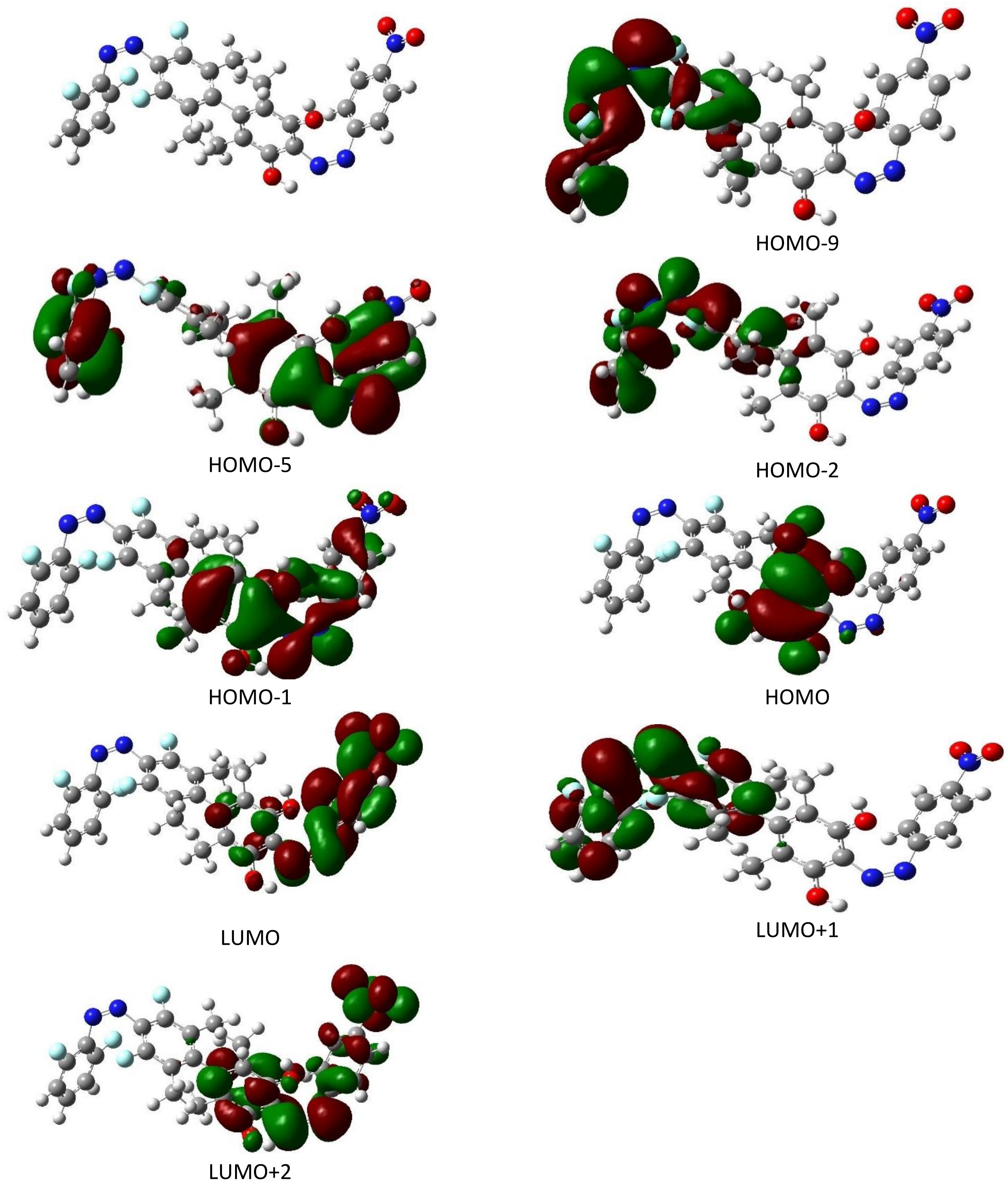

FIGURE S7: Shape of the molecular orbitals involved in the relevant excited electronic states of the ZZ isomer of dimer 2. The draws correspond to an iso-level of 0.02 a.u. of electronic density. The left top corner of the Figure depicts the molecular structure of the ZZ isomer.

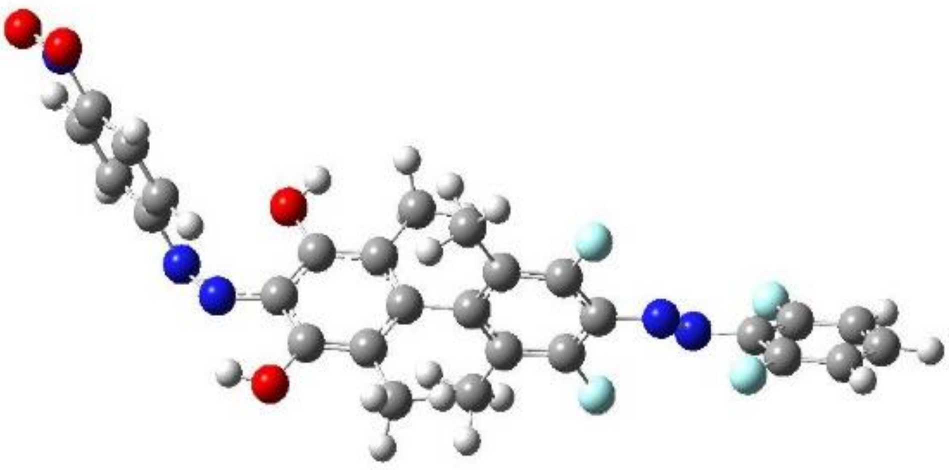

EE⟷ Z_PE_F

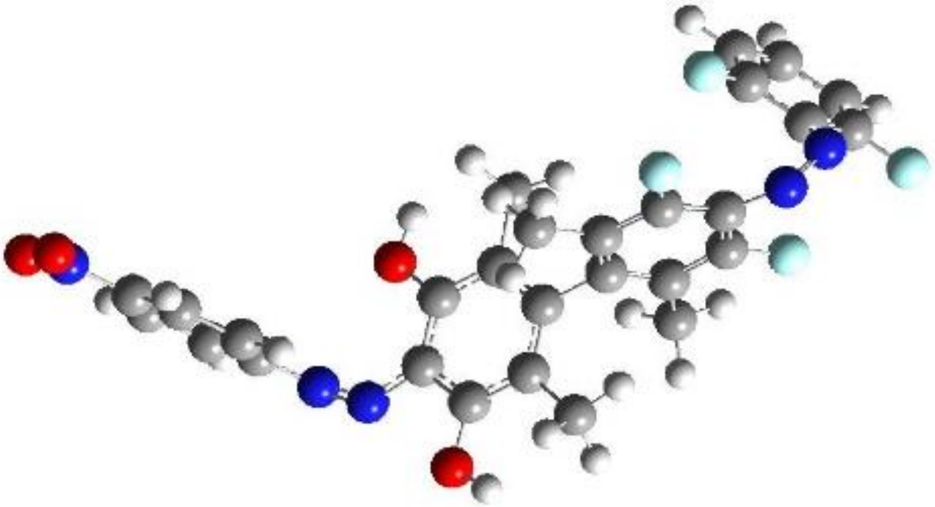

E_PZ_F⟷ ZZ

FIGURE S8: Approximate geometries of the conical intersections localized for dimer 2.

Table S1. Relative enthalpies and Gibbs free energies (kcal/mol) for the stationary points of dimer 1.

Isomer	ΔH	ΔG
EE	0.00	0.00
EZ_F	7.51	8.25
ZE_F	12.89	13.37
ZZ	20.40	22.36
TS EE ↔ EZ_F	37.37	36.53
TS EE ↔ ZE_F	38.89	39.71
TS ZZ ↔ EZ_F	46.41	48.18
TS ZZ ↔ ZE_F	50.22	49.64

Table S2. Comparison of the relative enthalpies and Gibbs free energies (kcal/mol) for the stationary points of dimer 2.

Isomer	ΔH	ΔG
EE	0.00	0.00
E_PZ_F	7.20	8.94
Z_PE_F	6.77	8.56
ZZ	13.91	15.42
TS EE ↔ E_PZ_F	37.21	37.32
TS EE ↔ Z_PE_F	28.19	29.96
TS ZZ ↔ E_PZ_F	31.10	35.41
TS ZZ ↔ Z_PE_F	43.86	44.56

Table S3. Comparison of the relative energies (kcal/mol) of the stationary points of dimer 1 in the ground electronic state calculated using two different basis sets.

Isomer	6-311+G(d,p)	DEF2-TZVP
EE	0.00	0.00
EZ$_F$	7.54	7.59
ZE$_F$	13.01	12.86
ZZ	20.54	20.45
TS EE ↔ EZ$_F$	38.67	38.19
TS EE ↔ ZE$_F$	40.33	39.54
TS ZZ ↔ EZ$_F$	47.85	47.12
TS ZZ ↔ ZE$_F$	51.65	51.04

Table S4. Comparison of the results of the relevant excited electronic states of dimer 1 calculated using two different basis sets.

		6-311+G(d,p)			DEF2-TZVP		
Isomer	State	E(eV)	λ(nm)	f	E(eV)	λ(nm)	f
EE	S_1	2.67	464	0.10	2.66	466	0.09
	S_2	2.75	451	0.00	2.73	454	0.00
	S_3	3.74	331	1.61	3.78	328	1.69
	S_4	4.10	302	0.78	4.11	301	0.76
EZ$_F$	S_1	2.75	451	0.00	2.73	454	0.00
	S_2	2.89	430	0.06	2.89	429	0.05
	S_3	3.75	330	1.29	3.79	327	1.33
	S_4	4.32	287	0.08	4.36	284	0.08
ZE$_F$	S_1	2.67	465	0.09	2.66	466	0.08
	S_2	2.78	445	0.05	2.79	444	0.04
	S_3	4.06	305	1.33	4.08	304	1.36
	S_4	4.47	277	0.07	4.49	276	0.07
	S_5	4.47	277	0.03	4.49	276	0.02
	S_6	4.48	276	0.16	4.55	273	0.17
ZZ	S_1	2.78	445	0.05	2.79	444	0.05
	S_2	2.88	430	0.05	2.89	429	0.05
	S_3	4.46	278	0.36	4.51	275	0.32
	S_4	4.50	275	0.02	4.55	272	0.06
	S_5	4.57	271	0.12	4.63	268	0.11

Table S5: Dimer 1. Cartesian coordinates of the EE isomer in Å.

7	-5.613536	-0.461604	-0.243803
7	-6.267952	0.515739	0.148340
6	-7.679887	0.397480	0.027748
6	-8.414097	1.478146	0.506353
6	-8.332988	-0.704275	-0.527813
6	-9.800458	1.462671	0.436370
1	-7.884401	2.322093	0.931088
6	-9.715273	-0.712723	-0.595940
1	-7.751962	-1.537085	-0.899411
6	-10.451997	0.367739	-0.114572
1	-10.369984	2.304343	0.810716
1	-10.226410	-1.565101	-1.027017
1	-11.533674	0.352475	-0.171861
6	-4.201255	-0.342097	-0.126847
6	-3.545312	0.783453	0.363673
6	-3.472521	-1.446599	-0.543733
6	-2.161099	0.808776	0.440588
1	-4.124393	1.639023	0.683959
6	-2.083018	-1.452399	-0.478171
1	-4.006088	-2.310111	-0.922717
6	-1.432455	-0.316570	0.017145
6	0.058287	-0.298684	0.096876
6	0.813640	0.144245	-0.985216
6	0.705921	-0.732463	1.250355
6	2.188384	0.166609	-0.898972
1	0.342642	0.471613	-1.903046
6	2.080672	-0.712630	1.293348
1	0.153259	-1.086568	2.110560
6	2.877206	-0.252370	0.243278
7	4.271489	-0.338183	0.413886
7	4.943330	0.463294	-0.258748
6	6.339928	0.328320	-0.130982
6	7.048237	-0.854148	0.105385

6	7.111763	1.471701	-0.347001
6	8.425583	-0.895827	0.141219
6	8.487360	1.474803	-0.300744
6	9.142013	0.276387	-0.053475
1	8.918733	-1.842501	0.316734
1	9.024887	2.399921	-0.458182
1	10.223244	0.254138	-0.017604
6	-1.312984	-2.663362	-0.935182
1	-0.643096	-2.420288	-1.762882
1	-0.694943	-3.069075	-0.131407
1	-1.992292	-3.447189	-1.268955
6	-1.460977	2.032222	0.971844
1	-0.872551	1.799236	1.862073
1	-0.772605	2.448940	0.233386
1	-2.183325	2.803989	1.235863
9	2.692783	-1.139509	2.408963
9	2.874996	0.561910	-1.979734
9	6.471428	2.626772	-0.590414
9	6.379862	-2.007286	0.253161

Table S6: Dimer 1. Cartesian coordinates of the EZ_F isomer in Å.

7	-4.990845	0.437981	0.302187
7	-5.521327	-0.598450	-0.124019
6	-6.934418	-0.668179	0.018359
6	-7.533476	-1.808112	-0.508513
6	-7.712237	0.310536	0.639773
6	-8.909000	-1.974061	-0.421996
1	-6.908346	-2.554290	-0.983572
6	-9.082849	0.138012	0.724400
1	-7.235151	1.190459	1.048954
6	-9.684564	-1.001669	0.194340
1	-9.373386	-2.861362	-0.834248
1	-9.690445	0.894162	1.206800
1	-10.758108	-1.128326	0.265058
6	-3.576785	0.504352	0.166456
6	-2.786286	-0.525030	-0.336708
6	-2.992590	1.692598	0.580553
6	-1.411782	-0.369244	-0.432152
1	-3.252928	-1.448819	-0.651095
6	-1.616800	1.880078	0.495674
1	-3.629161	2.477713	0.970925
6	-0.830246	0.840790	-0.014203
6	0.648985	1.017913	-0.116363
6	1.469321	0.715275	0.966205
6	1.218753	1.493621	-1.296002
6	2.832810	0.872065	0.846277
1	1.060126	0.360892	1.903329
6	2.582989	1.652782	-1.366387
1	0.610937	1.741833	-2.156251
6	3.436617	1.325479	-0.319919
7	4.820778	1.658037	-0.421903
7	5.713655	0.835704	-0.192582
6	5.436134	-0.547508	0.027435

6	4.813330	-1.366013	-0.907924
6	5.984918	-1.177286	1.138870
6	4.705862	-2.730430	-0.745139
6	5.885197	-2.532978	1.352215
6	5.240112	-3.308658	0.397135
1	4.214919	-3.318766	-1.508527
1	6.309708	-2.964997	2.248279
1	5.158116	-4.377532	0.543185
6	-1.005377	3.180138	0.947533
1	-0.316695	3.027631	1.781494
1	-0.436362	3.653172	0.144445
1	-1.778437	3.876404	1.271412
6	-0.565830	-1.490769	-0.975956
1	-0.034892	-1.186426	-1.880642
1	0.189206	-1.806440	-0.252721
1	-1.183873	-2.354086	-1.220660
9	3.134127	2.117893	-2.499494
9	3.614918	0.611567	1.909768
9	6.610940	-0.408014	2.045231
9	4.331371	-0.798818	-2.029474

Table S7: Dimer 1. Cartesian coordinates of the ZE_F isomer in Å.

7	-6.065481	-1.686906	0.210317
7	-6.940016	-0.815249	0.160156
6	-6.656721	0.569097	-0.096648
6	-7.258569	1.501753	0.740538
6	-5.951907	0.981982	-1.223603
6	-7.095345	2.855964	0.488873
1	-7.840041	1.158652	1.587740
6	-5.822251	2.338024	-1.485800
1	-5.519681	0.251209	-1.894665
6	-6.379228	3.276989	-0.625491
1	-7.543437	3.583495	1.154530
1	-5.281911	2.662021	-2.366948
1	-6.267528	4.334418	-0.831429
6	-4.660279	-1.393436	0.169088
6	-4.063559	-0.537565	1.084061
6	-3.885881	-2.125643	-0.717557
6	-2.682458	-0.378445	1.095714
1	-4.670546	0.002375	1.799672
6	-2.506148	-1.961539	-0.750169
1	-4.368001	-2.819941	-1.395667
6	-1.907934	-1.084052	0.164661
6	-0.425043	-0.909925	0.158529
6	0.158647	0.107643	-0.591104
6	0.387967	-1.765228	0.897383
6	1.526512	0.270808	-0.580120
1	-0.442345	0.778814	-1.190895
6	1.751174	-1.583063	0.870091
1	-0.028096	-2.568613	1.490917
6	2.377090	-0.560217	0.155290
7	3.783659	-0.524997	0.188272
7	4.293755	0.589245	-0.023783
6	5.701626	0.612191	-0.049416

6	6.552438	-0.424868	-0.445756
6	6.320567	1.821890	0.273439
6	7.921737	-0.276830	-0.501602
6	7.684594	2.003893	0.247675
6	8.484805	0.939778	-0.143695
1	8.528077	-1.111600	-0.826709
1	8.101083	2.961792	0.527876
1	9.559534	1.061275	-0.175737
6	-1.683460	-2.734515	-1.746963
1	-1.106232	-2.065303	-2.388656
1	-0.970226	-3.394071	-1.247878
1	-2.323037	-3.345983	-2.382750
6	-2.046742	0.547509	2.099250
1	-1.312990	0.023268	2.715162
1	-1.522047	1.367994	1.604691
1	-2.799759	0.977223	2.759199
9	2.523395	-2.417552	1.583623
9	2.045924	1.237848	-1.348996
9	5.539616	2.848589	0.646497
9	6.030393	-1.596092	-0.837036

Table S8: Dimer 1. Cartesian coordinates of the ZZ isomer in Å.

7	5.505175	1.507038	0.374869
7	6.275430	0.541219	0.340504
6	5.853277	-0.790142	0.006375
6	6.287897	-1.816653	0.837282
6	5.181873	-1.076754	-1.178670
6	5.987589	-3.132421	0.517250
1	6.847726	-1.574982	1.732708
6	4.915397	-2.397901	-1.507271
1	4.880956	-0.276613	-1.842346
6	5.303031	-3.426214	-0.656189
1	6.304571	-3.930950	1.176840
1	4.400914	-2.624199	-2.433268
1	5.084936	-4.455166	-0.914500
6	4.081566	1.382287	0.234278
6	3.332030	0.567417	1.070804
6	3.455779	2.234425	-0.662374
6	1.943968	0.572477	0.993214
1	3.825443	-0.068746	1.794579
6	2.071460	2.233360	-0.786918
1	4.057534	2.895247	-1.275142
6	1.317536	1.398136	0.049453
6	-0.172354	1.402253	-0.052797
6	-0.814931	0.599803	-0.990743
6	-0.931928	2.216373	0.785843
6	-2.190785	0.609324	-1.061335
1	-0.257578	-0.031749	-1.670208
6	-2.302102	2.209090	0.669227
1	-0.466217	2.856606	1.523586
6	-2.979953	1.393659	-0.229102
7	-4.388755	1.556160	-0.386183
7	-5.170494	0.600108	-0.353059
6	-4.751124	-0.715297	0.009577

6	-4.184211	-1.032356	1.238728
6	-5.101650	-1.783422	-0.808743
6	-3.942849	-2.331345	1.631339
6	-4.862501	-3.094492	-0.466498
6	-4.279262	-3.361940	0.765719
1	-3.504031	-2.519485	2.601904
1	-5.133705	-3.883922	-1.154257
1	-4.090329	-4.386824	1.056378
6	1.410599	3.134825	-1.796162
1	0.906904	2.555817	-2.573703
1	0.655130	3.770022	-1.329606
1	2.146271	3.777531	-2.278962
6	1.143884	-0.309161	1.916037
1	0.515944	0.282828	2.585691
1	0.480210	-0.971991	1.356891
1	1.802480	-0.924672	2.528061
9	-3.035317	2.999248	1.470733
9	-2.799952	-0.142033	-1.996888
9	-5.671930	-1.500776	-1.991992
9	-3.897252	-0.026706	2.086202

Table S9: Dimer 1. Cartesian coordinates of the EE to EZ_F transition state in Å.

7	5.395990	0.343137	-0.476921
7	6.065879	-0.551784	0.059201
6	7.456607	-0.538872	-0.236712
6	8.206165	-1.543934	0.366541
6	8.075677	0.395901	-1.068661
6	9.574090	-1.620693	0.142776
1	7.702795	-2.257105	1.007824
6	9.439742	0.313704	-1.287544
1	7.483087	1.172694	-1.532011
6	10.191740	-0.692362	-0.684060
1	10.155695	-2.403821	0.613243
1	9.924647	1.036710	-1.932328
1	11.258973	-0.749037	-0.861113
6	4.004923	0.329565	-0.180558
6	3.386878	-0.603188	0.647464
6	3.257548	1.332653	-0.780439
6	2.021339	-0.537581	0.878892
1	3.980326	-1.380538	1.109458
6	1.886020	1.425968	-0.568195
1	3.762025	2.046455	-1.420647
6	1.274598	0.483102	0.265487
6	-0.196508	0.562129	0.506885
6	-1.079020	-0.117983	-0.330798
6	-0.693493	1.315317	1.566773
6	-2.431668	-0.035125	-0.099510
1	-0.722354	-0.712122	-1.162038
6	-2.053622	1.373235	1.767899
1	-0.034890	1.854376	2.234787
6	-2.971226	0.709343	0.952568
7	-4.348230	0.864334	1.297001
7	-5.185716	0.324634	0.602125
6	-6.118367	-0.260725	-0.131190

6	-6.799023	0.410410	-1.159475
6	-6.493072	-1.599719	0.062765
6	-7.768435	-0.186929	-1.926924
6	-7.468109	-2.217840	-0.680762
6	-8.115471	-1.512527	-1.689341
1	-8.245724	0.396080	-2.704187
1	-7.706888	-3.251458	-0.465243
1	-8.881136	-1.990544	-2.283890
6	1.093325	2.522786	-1.229009
1	0.318765	2.115977	-1.882918
1	0.591888	3.152250	-0.490693
1	1.743344	3.158267	-1.829637
6	1.361684	-1.551301	1.776671
1	0.879536	-1.072034	2.631605
1	0.589085	-2.111473	1.245266
1	2.094708	-2.262359	2.156133
9	-2.518880	2.099678	2.791038
9	-3.257989	-0.700503	-0.917528
9	-5.847051	-2.271023	1.046864
9	-6.447554	1.701117	-1.375120

Table S10: Dimer 1. Cartesian coordinates of the EE to ZE$_F$ transition state in Å.

7	5.684121	1.531366	-0.645124
7	6.470389	0.653235	-0.357320
6	7.330641	-0.307965	-0.034929
6	7.743451	-1.253480	-1.000215
6	7.857285	-0.386549	1.273881
6	8.654878	-2.232908	-0.648393
1	7.342457	-1.199713	-2.003717
6	8.766387	-1.382169	1.583690
1	7.543995	0.333819	2.017978
6	9.179114	-2.315588	0.637870
1	8.959893	-2.948506	-1.403712
1	9.159769	-1.425065	2.593185
1	9.890912	-3.088365	0.896855
6	4.276336	1.246503	-0.466555
6	3.791150	0.028798	-0.011884
6	3.418216	2.283699	-0.784556
6	2.424749	-0.162451	0.130627
1	4.480402	-0.771153	0.229862
6	2.042417	2.123846	-0.651099
1	3.834109	3.220016	-1.137510
6	1.554868	0.894137	-0.192112
6	0.081805	0.700085	-0.044340
6	-0.674711	0.237754	-1.117107
6	-0.547464	0.988498	1.163139
6	-2.032131	0.054019	-0.969881
1	-0.219403	0.020420	-2.074653
6	-1.907427	0.809084	1.267435
1	0.006836	1.352795	2.017964
6	-2.702593	0.324403	0.227109
7	-4.086451	0.240179	0.468498
7	-4.704046	-0.577324	-0.236381
6	-6.096994	-0.614834	-0.033385

6	-6.917672	0.445483	0.365414
6	-6.748849	-1.811210	-0.340902
6	-8.286705	0.321916	0.467491
6	-8.110687	-1.979493	-0.233938
6	-8.878725	-0.898486	0.175146
1	-8.870962	1.180527	0.770138
1	-8.550604	-2.938784	-0.470110
1	-9.951921	-1.006224	0.261621
6	1.114834	3.258049	-0.997772
1	0.432948	2.981215	-1.804691
1	0.499870	3.544446	-0.142072
1	1.680126	4.132898	-1.317327
6	1.895857	-1.483718	0.622553
1	1.332675	-1.364966	1.550661
1	1.220756	-1.937332	-0.106202
1	2.712888	-2.179988	0.808444
9	-2.502543	1.096684	2.435259
9	-2.724846	-0.354575	-2.041226
9	-5.999861	-2.851049	-0.741839
9	-6.375086	1.646410	0.612457

Table S11: Dimer 1. Cartesian coordinates of the ZZ to EZ_F transition state in Å.

7	-5.078680	-1.550918	-0.701194
7	-5.773390	-0.589189	-0.446926
6	-6.532543	0.466757	-0.170394
6	-7.084981	0.633236	1.119321
6	-6.811074	1.428276	-1.167517
6	-7.888041	1.729030	1.380176
1	-6.873578	-0.098826	1.887527
6	-7.619124	2.509407	-0.864330
1	-6.389640	1.307086	-2.156708
6	-8.168112	2.679163	0.402955
1	-8.303433	1.838572	2.375708
1	-7.822229	3.235801	-1.643284
1	-8.798173	3.530824	0.623494
6	-3.654166	-1.416963	-0.483011
6	-3.057657	-0.264852	0.008853
6	-2.900073	-2.532291	-0.800346
6	-1.683154	-0.220040	0.189469
1	-3.666677	0.597681	0.251086
6	-1.518728	-2.517765	-0.633403
1	-3.401494	-3.414566	-1.180333
6	-0.918464	-1.354411	-0.136426
6	0.562767	-1.316079	0.050862
6	1.391919	-0.941180	-1.001615
6	1.124716	-1.663375	1.277790
6	2.755216	-0.902440	-0.806856
1	0.990938	-0.681191	-1.972604
6	2.492000	-1.628686	1.422860
1	0.509834	-1.962517	2.116543
6	3.349549	-1.225933	0.406494
7	4.758400	-1.364701	0.589028
7	5.544435	-0.437020	0.371768
6	5.097220	0.888505	0.086803

6	4.323309	1.643866	0.960510
6	5.616312	1.552511	-1.018978
6	4.046776	2.976609	0.743626
6	5.350646	2.875951	-1.285946
6	4.559928	3.586683	-0.391782
1	3.443592	3.516928	1.460659
1	5.761549	3.333992	-2.175393
1	4.346254	4.630441	-0.580136
6	-0.704881	-3.734707	-0.985016
1	-0.001781	-3.521839	-1.793211
1	-0.117948	-4.081331	-0.132084
1	-1.352283	-4.549901	-1.306516
6	-1.033939	1.028351	0.725466
1	-0.532930	0.837196	1.676967
1	-0.278089	1.410445	0.036202
1	-1.775895	1.810088	0.884623
9	3.037814	-1.971429	2.600988
9	3.551844	-0.573883	-1.839913
9	6.386138	0.847393	-1.864653
9	3.861327	1.050828	2.077059

Table S12: Dimer 1. Cartesian coordinates of the ZZ to ZE_F transition state in Å.

7	5.965657	-1.358786	0.498934
7	6.746251	-0.407377	0.387286
6	6.310444	0.951375	0.224971
6	6.916929	1.693335	-0.782261
6	5.445206	1.560857	1.128773
6	6.601930	3.036094	-0.929501
1	7.621440	1.210017	-1.448320
6	5.162073	2.912196	0.993986
1	5.007546	0.987722	1.935710
6	5.726266	3.648881	-0.040821
1	7.055086	3.609091	-1.729174
1	4.496067	3.391179	1.701352
1	5.494975	4.701747	-0.145659
6	4.545381	-1.233459	0.327527
6	3.991912	-0.729269	-0.840861
6	3.736496	-1.789493	1.306574
6	2.614283	-0.744970	-1.026378
1	4.632183	-0.330333	-1.617369
6	2.354116	-1.786657	1.163922
1	4.191585	-2.216951	2.192240
6	1.798468	-1.262467	-0.011220
6	0.316354	-1.269731	-0.190439
6	-0.449346	-0.204378	0.281085
6	-0.308348	-2.338702	-0.826996
6	-1.813161	-0.221929	0.108931
1	0.007672	0.639638	0.781026
6	-1.675893	-2.322408	-0.979050
1	0.257448	-3.180809	-1.202903
6	-2.478199	-1.274793	-0.524765
7	-3.880824	-1.409361	-0.754792
7	-4.616578	-0.507664	-0.407206
6	-5.440467	0.459653	-0.038895

6	-6.006706	0.515717	1.244422
6	-5.806289	1.504177	-0.902686
6	-6.869717	1.509061	1.637140
6	-6.664130	2.511470	-0.535223
6	-7.207210	2.520977	0.745005
1	-7.268162	1.480884	2.643279
1	-6.898752	3.281586	-1.258945
1	-7.884861	3.308109	1.044071
6	1.486394	-2.357458	2.254169
1	0.827360	-1.595409	2.676431
1	0.848909	-3.159925	1.877158
1	2.097159	-2.759850	3.061693
6	2.026648	-0.204766	-2.303691
1	1.494809	-0.982491	-2.856323
1	1.310266	0.595179	-2.105140
1	2.808363	0.193410	-2.949744
9	-2.264444	-3.356395	-1.591987
9	-2.526030	0.812852	0.573702
9	-5.265796	1.477990	-2.145152
9	-5.663869	-0.477820	2.099967

Table S13: Dimer 2. Cartesian coordinates of the EE isomer in Å.

7	5.424711	0.263819	0.474173
7	6.105904	-0.314035	-0.390081
6	7.500288	-0.152855	-0.261862
6	8.307117	-1.181427	-0.751676
6	8.169869	0.971970	0.228832
6	9.682649	-1.136606	-0.733130
6	9.545226	1.063298	0.247445
6	10.298867	-0.000862	-0.226673
1	10.249675	-1.976612	-1.110492
1	10.007766	1.963874	0.628500
1	11.379104	0.058438	-0.206447
6	4.029058	0.153450	0.307613
6	3.341974	0.105785	-0.902506
6	3.245405	0.181238	1.459095
6	1.960602	0.062966	-0.990374
6	1.865723	0.128404	1.453595
6	1.231723	0.064604	0.204329
6	-0.263446	-0.001621	0.156639
6	-0.995280	1.180865	0.012743
6	-0.889235	-1.248768	0.260104
6	-2.389153	1.107368	-0.044919
6	-2.280384	-1.288368	0.222308
6	-3.053655	-0.127245	0.057283
7	-4.440825	-0.318317	0.110534
7	-5.161291	0.519147	-0.460942
6	-6.554643	0.280681	-0.307106
6	-7.097878	-0.507276	0.708631
6	-7.383514	0.919493	-1.225506
6	-8.466488	-0.672133	0.790074
6	-8.754928	0.753213	-1.158114
6	-9.272821	-0.042319	-0.148648
1	-8.912168	-1.274481	1.568340

1	-9.414586	1.231548	-1.867129
6	1.097963	0.126426	2.746913
1	0.230696	0.783546	2.685500
1	0.733438	-0.877071	2.980891
1	1.723488	0.455043	3.574088
6	1.312908	0.036801	-2.347867
1	0.303175	-0.364442	-2.292620
1	1.250937	1.044842	-2.767037
1	1.891163	-0.570195	-3.043827
1	-6.442623	-0.977762	1.427993
1	-6.939290	1.539456	-1.993237
7	-10.725885	-0.217260	-0.062319
6	-0.129337	-2.542209	0.402400
1	-0.362248	-3.235825	-0.411883
1	0.945435	-2.380357	0.382265
1	-0.359584	-3.045363	1.347370
6	-0.344868	2.538027	-0.075452
1	-0.652635	3.188470	0.750970
1	0.738708	2.466452	-0.027941
1	-0.594886	3.045316	-1.012450
8	-11.168724	-0.917750	0.831059
8	-11.422791	0.344595	-0.889079
8	-2.953280	-2.460905	0.312918
1	-2.338862	-3.186832	0.465677
8	-3.131042	2.232715	-0.154615
1	-2.564424	3.006998	-0.062706
9	7.702530	-2.275032	-1.244100
9	7.462384	2.029992	0.651499
9	4.038828	0.157923	-2.051326
9	3.888888	0.236545	2.641408

Table S14: Dimer 2. Cartesian coordinates of the EZ_F isomer in Å.

7	5.880986	-1.579107	0.067769
7	6.760589	-0.716014	-0.016503
6	6.465557	0.680514	0.035456
6	6.982926	1.515758	-0.947971
6	5.857183	1.299775	1.121159
6	6.866135	2.886093	-0.899562
6	5.733352	2.668927	1.221027
6	6.235630	3.459375	0.197502
1	7.266324	3.484867	-1.706491
1	5.254712	3.096306	2.091804
1	6.140556	4.535442	0.257413
6	4.491171	-1.252549	0.061044
6	3.863077	-0.595518	-0.985237
6	3.673134	-1.783734	1.047378
6	2.492110	-0.419334	-1.058041
6	2.299901	-1.638648	1.056401
6	1.720356	-0.943930	-0.015443
6	0.233437	-0.769554	-0.046236
6	-0.337921	0.325423	0.609564
6	-0.546595	-1.707820	-0.730657
6	-1.724271	0.490410	0.563000
6	-1.926967	-1.525710	-0.746712
6	-2.539522	-0.428302	-0.119918
7	-3.940712	-0.419711	-0.153629
7	-4.511818	0.681673	-0.066477
6	-5.931414	0.594059	-0.042721
6	-6.630844	-0.571405	0.274260
6	-6.615085	1.773826	-0.323842
6	-8.011504	-0.559170	0.289256
6	-7.998202	1.794353	-0.319988
6	-8.672835	0.623884	-0.012574
1	-8.576264	-1.447011	0.534203

1	-8.547657	2.696861	-0.544151
6	1.476409	-2.207606	2.178626
1	0.983384	-1.411445	2.740313
1	0.696124	-2.865085	1.792155
1	2.094113	-2.776837	2.869391
6	1.886485	0.292029	-2.236769
1	1.521602	-0.425750	-2.976288
1	1.040786	0.905413	-1.928413
1	2.618252	0.930551	-2.727769
1	-6.085621	-1.473686	0.512417
1	-6.049969	2.668649	-0.549788
7	-10.139022	0.637040	0.004050
6	0.037045	-2.901633	-1.441450
1	-0.201569	-2.891350	-2.509785
1	1.120602	-2.928235	-1.357254
1	-0.337142	-3.842007	-1.023325
6	0.479796	1.341293	1.366238
1	0.229250	1.352975	2.432886
1	1.543712	1.130243	1.295870
1	0.326357	2.351978	0.975777
8	-2.744019	-2.391128	-1.395011
1	-2.232760	-3.119394	-1.763749
8	-2.316897	1.516514	1.215250
1	-1.669240	1.974719	1.762366
8	-10.707777	1.680735	-0.264482
8	-10.720231	-0.396805	0.284044
9	4.280059	-2.445328	2.052731
9	5.406460	0.525518	2.125880
9	7.596848	0.940012	-1.995597
9	4.636010	-0.141728	-1.994701

Table S15: Dimer 2. Cartesian coordinates of the ZE$_F$ isomer in Å.

7	-4.529353	0.479467	-0.478424
7	-5.321907	0.289283	0.460447
6	-6.647482	0.702696	0.221567
6	-7.414804	1.045320	1.336870
6	-7.310698	0.727864	-1.009477
6	-8.736157	1.422788	1.258955
6	-8.637436	1.083186	-1.127088
6	-9.344854	1.439939	0.011958
1	-9.268294	1.693787	2.160587
1	-9.099812	1.072951	-2.104999
1	-10.384114	1.729067	-0.072929
6	-3.218097	0.010161	-0.265530
6	-2.841204	-1.095372	0.493720
6	-2.194345	0.660993	-0.952605
6	-1.532121	-1.535478	0.596746
6	-0.866221	0.289970	-0.889573
6	-0.548277	-0.823664	-0.098568
6	0.881572	-1.260326	-0.013506
6	1.719330	-0.662580	0.938574
6	1.335216	-2.264692	-0.875727
6	3.053139	-1.061669	0.995437
6	2.667114	-2.649859	-0.774940
6	3.552439	-2.016407	0.104963
7	4.846911	-2.595476	0.135798
7	5.912524	-1.972398	0.243342
6	6.012947	-0.554784	0.168052
6	5.484706	0.148356	-0.912457
6	6.823794	0.081741	1.104380
6	5.731420	1.503807	-1.031303
6	7.054763	1.438754	1.004875
6	6.500429	2.130083	-0.063214
1	5.336242	2.070806	-1.861586

1	7.663204	1.957757	1.730978
6	0.181890	1.064020	-1.640934
1	0.820767	1.622010	-0.952345
1	0.824832	0.392598	-2.211004
1	-0.272231	1.773773	-2.328612
6	-1.231557	-2.757577	1.420838
1	-1.411613	-3.668057	0.842639
1	-0.192129	-2.767197	1.742049
1	-1.869256	-2.799997	2.303145
1	4.898507	-0.360991	-1.665189
1	7.256799	-0.492889	1.912376
7	6.752201	3.567787	-0.182252
9	-6.820605	1.021715	2.541084
9	-3.790323	-1.813508	1.119268
9	-2.536435	1.727866	-1.700652
9	-6.662044	0.345597	-2.119100
6	1.237836	0.373924	1.921513
1	0.198669	0.640576	1.745277
1	1.301595	0.011267	2.952940
1	1.822693	1.296682	1.853935
6	0.464211	-2.956447	-1.888806
1	-0.527202	-2.511634	-1.938726
1	0.912263	-2.906396	-2.883435
1	0.350407	-4.016023	-1.645771
8	6.263179	4.161597	-1.127610
8	7.437229	4.104171	0.671234
8	3.112552	-3.644108	-1.579744
1	4.015406	-3.874101	-1.310418
8	3.907382	-0.585394	1.932921
1	3.457245	0.021717	2.530583

Table S16: Dimer 2. Cartesian coordinates of the ZZ isomer in Å.

7	-4.461232	2.409886	0.537882
7	-5.621169	2.017308	0.375572
6	-5.922103	0.713889	-0.124027
6	-6.837524	-0.076060	0.561968
6	-5.512010	0.252416	-1.369471
6	-7.290782	-1.282415	0.079423
6	-5.953022	-0.940536	-1.900840
6	-6.840674	-1.710371	-1.163215
1	-7.984277	-1.866662	0.668907
1	-5.604015	-1.247475	-2.877576
1	-7.191071	-2.652597	-1.563307
6	-3.336394	1.542384	0.398218
6	-3.146759	0.402006	1.160187
6	-2.268317	1.950801	-0.389274
6	-1.981125	-0.345584	1.137836
6	-1.080091	1.255324	-0.479063
6	-0.951222	0.094779	0.301380
6	0.326277	-0.682352	0.225427
6	1.369440	-0.378150	1.106806
6	0.422676	-1.708571	-0.723165
6	2.557022	-1.101884	1.003167
6	1.611276	-2.424932	-0.780307
6	2.708475	-2.088915	0.026488
7	3.806871	-2.975487	-0.107010
7	5.003779	-2.655650	-0.079136
6	5.466991	-1.310363	-0.090727
6	5.042048	-0.407878	-1.063375
6	6.504153	-0.983456	0.779241
6	5.626219	0.843328	-1.135850
6	7.075190	0.271916	0.728241
6	6.623145	1.168580	-0.229646
1	5.317059	1.559676	-1.883065

1	7.866651	0.554743	1.406776
6	0.023329	1.736966	-1.380173
1	0.934795	1.924789	-0.809964
1	0.261497	0.986627	-2.136777
1	-0.257636	2.656428	-1.888498
6	-1.870511	-1.558679	2.019829
1	-1.089518	-2.229343	1.667870
1	-1.627091	-1.269711	3.045814
1	-2.812586	-2.105732	2.050347
1	4.270873	-0.684259	-1.769368
1	6.846020	-1.715245	1.499134
7	7.234159	2.497696	-0.298523
6	-0.718755	-2.070892	-1.635659
1	-1.472560	-2.664030	-1.109473
1	-1.218365	-1.181848	-2.021635
1	-0.366218	-2.660365	-2.479554
6	1.262461	0.682353	2.172055
1	0.312870	1.209004	2.116664
1	1.329944	0.252682	3.177096
1	2.052825	1.433164	2.074476
8	3.588198	-0.916161	1.861344
1	3.363134	-0.263075	2.533235
8	1.720813	-3.453537	-1.655864
1	2.558271	-3.911088	-1.481141
8	6.835162	3.271252	-1.151681
8	8.111728	2.769234	0.502473
9	-7.266055	0.361981	1.757962
9	-4.679676	1.024841	-2.092265
9	-2.440829	3.074260	-1.114329
9	-4.139121	0.026641	1.994802

Table S17: Dimer 2. Cartesian coordinates of the EE to E_PZ_F transition state in Å.

7	-5.508980	1.277198	0.668671
7	-6.319185	0.460878	0.277990
6	-7.224002	-0.409093	-0.136397
6	-7.765892	-0.371111	-1.431293
6	-7.707822	-1.439599	0.685722
6	-8.710734	-1.264314	-1.872660
6	-8.652140	-2.345830	0.270485
6	-9.164880	-2.264573	-1.019725
1	-9.081785	-1.167662	-2.885032
1	-8.976629	-3.110349	0.964870
1	-9.908790	-2.972601	-1.356640
6	-4.118020	1.003140	0.496755
6	-3.539274	-0.136300	-0.061144
6	-3.244041	1.991030	0.944457
6	-2.174731	-0.307866	-0.185938
6	-1.867991	1.888865	0.861591
6	-1.348184	0.722753	0.286216
6	0.136493	0.569917	0.169188
6	0.831431	-0.106242	1.175978
6	0.786570	1.107727	-0.946579
6	2.214078	-0.261141	1.049448
6	2.167905	0.955304	-1.033895
6	2.901706	0.262048	-0.058303
7	4.291949	0.250701	-0.243654
7	4.926878	-0.700250	0.244695
6	6.334581	-0.605096	0.066219
6	7.000846	0.593183	-0.193471
6	7.045441	-1.794269	0.203123
6	8.374275	0.595833	-0.337425
6	8.419973	-1.804006	0.049226
6	9.060846	-0.605114	-0.218916
1	8.914389	1.510328	-0.535310

1	8.989305	-2.717373	0.140188
6	-0.991649	3.004133	1.360619
1	-0.107983	2.610259	1.861253
1	-0.650221	3.628767	0.531142
1	-1.530420	3.642622	2.057649
6	-1.623382	-1.569079	-0.792065
1	-0.764330	-1.352511	-1.425883
1	-1.292395	-2.260139	-0.012525
1	-2.376633	-2.078208	-1.389627
1	6.436936	1.511773	-0.273451
1	6.507798	-2.707312	0.423082
7	10.518898	-0.602776	-0.374077
6	0.063927	1.830745	-2.053695
1	0.211711	1.335411	-3.018617
1	-1.008293	1.868051	-1.877934
1	0.407190	2.865753	-2.152497
6	0.155656	-0.668323	2.400638
1	0.521350	-0.193860	3.317994
1	-0.919383	-0.508825	2.375989
1	0.320327	-1.746197	2.492459
8	11.073484	0.462632	-0.579253
8	11.107465	-1.666568	-0.292324
8	2.865500	1.445630	-2.086700
1	2.280828	1.932768	-2.677271
8	2.927937	-0.878548	2.018086
1	2.366348	-1.044470	2.783599
9	-7.309755	0.609797	-2.248161
9	-7.195331	-1.503152	1.938979
9	-4.349179	-1.115917	-0.497468
9	-3.775428	3.099497	1.483733

Table S18: Dimer 2. Cartesian coordinates of the EE to Z_PE_F transition state in Å.

7	-5.103789	-0.575677	0.465850
7	-5.905324	-0.133991	-0.375131
6	-7.224600	-0.615185	-0.255375
6	-8.250110	0.217181	-0.707387
6	-7.613215	-1.882130	0.190050
6	-9.577932	-0.144994	-0.694103
6	-8.930079	-2.289426	0.201382
6	-9.912010	-1.410800	-0.233439
1	-10.326046	0.554952	-1.040460
1	-9.169603	-3.286500	0.545852
1	-10.949216	-1.718750	-0.218495
6	-3.769602	-0.150076	0.303342
6	-3.122949	0.098085	-0.902997
6	-2.985177	-0.057946	1.452415
6	-1.781242	0.432894	-0.993084
6	-1.650407	0.291939	1.446585
6	-1.057682	0.536718	0.198140
6	0.392259	0.909728	0.158483
6	1.349258	-0.107877	0.064385
6	0.744320	2.262567	0.224701
6	2.694525	0.250612	0.037488
6	2.097165	2.592297	0.191820
6	3.088062	1.598493	0.100930
7	4.409257	2.082294	0.083076
7	5.361399	1.336504	0.012262
6	6.358761	0.511876	-0.062086
6	6.962136	-0.006498	1.126394
6	6.887290	0.124085	-1.333086
6	8.029902	-0.851462	1.032333
6	7.955999	-0.722679	-1.393758
6	8.542208	-1.219563	-0.220403
1	8.486873	-1.246524	1.928857

1	8.357062	-1.018807	-2.352976
6	-0.877304	0.396705	2.732567
1	-0.055067	-0.321336	2.751671
1	-0.443832	1.391889	2.846064
1	-1.516396	0.203120	3.590712
6	-1.182959	0.652399	-2.355616
1	-0.148133	0.978804	-2.286486
1	-1.212024	-0.268370	-2.942524
1	-1.745400	1.406115	-2.909456
1	6.560214	0.279365	2.088296
1	6.429241	0.509277	-2.233283
7	9.647601	-2.101156	-0.301130
6	-0.268415	3.373281	0.327015
1	-0.213272	4.048422	-0.532972
1	-1.283904	2.987561	0.362768
1	-0.120221	3.969762	1.232925
6	0.992329	-1.569714	-0.013145
1	1.427226	-2.135772	0.816860
1	-0.083086	-1.720872	0.028733
1	1.341325	-2.019998	-0.947898
8	10.150134	-2.529977	0.741081
8	10.081250	-2.419134	-1.411757
8	2.511055	3.874151	0.246045
1	1.754497	4.468473	0.303524
8	3.669196	-0.675527	-0.052100
1	3.292940	-1.562890	-0.085693
9	-7.917663	1.438512	-1.156252
9	-6.677699	-2.763497	0.572637
9	-3.801900	-0.044042	-2.053957
9	-3.589790	-0.305739	2.630020

Table S19: Dimer 2. Cartesian coordinates of the ZZ to E_PZ_F transition state in Å.

7	-5.301767	-2.059818	0.822765
7	-6.348879	-1.644345	0.316322
6	-6.431610	-0.369947	-0.323049
6	-6.984787	-0.288278	-1.595905
6	-6.178370	0.833971	0.324091
6	-7.230130	0.909886	-2.226728
6	-6.425107	2.057247	-0.261145
6	-6.945924	2.087423	-1.546621
1	-7.638420	0.912119	-3.228282
1	-6.213692	2.962367	0.292020
1	-7.138715	3.039612	-2.022783
6	-4.057905	-1.376269	0.666431
6	-3.463491	-1.141098	-0.563106
6	-3.295192	-1.103991	1.792695
6	-2.187874	-0.622259	-0.703899
6	-2.021682	-0.573082	1.739075
6	-1.479934	-0.335134	0.467736
6	-0.100438	0.238694	0.366633
6	0.987831	-0.640007	0.232025
6	0.052761	1.628291	0.409387
6	2.264988	-0.099505	0.135963
6	1.342348	2.139280	0.310882
6	2.455821	1.291866	0.174591
7	3.668944	1.985820	0.091395
7	4.737634	1.427106	-0.024826
6	5.878613	0.817593	-0.144921
6	6.623375	0.443191	1.014466
6	6.413145	0.527397	-1.436892
6	7.834023	-0.173744	0.873191
6	7.626700	-0.090733	-1.545694
6	8.350977	-0.445406	-0.400187

1	8.400852	-0.458830	1.748377
1	8.034692	-0.312469	-2.521912
6	-1.267332	-0.263741	3.002526
1	-0.262463	-0.686403	2.968522
1	-1.164589	0.815473	3.137562
1	-1.780077	-0.665209	3.873644
6	-1.609846	-0.411104	-2.076282
1	-2.396905	-0.295796	-2.819154
1	-0.976888	0.474872	-2.100528
1	-0.996082	-1.265767	-2.373700
1	6.216704	0.655169	1.993255
1	5.847337	0.803045	-2.315778
7	9.610431	-1.088701	-0.529681
6	-1.089788	2.596799	0.548737
1	-1.129865	3.273531	-0.307999
1	-2.046486	2.084971	0.622014
1	-0.964148	3.219111	1.437786
6	0.837026	-2.139010	0.193038
1	-0.202559	-2.439243	0.295503
1	1.200178	-2.556777	-0.751342
1	1.388480	-2.619760	1.007416
8	3.364638	-0.867673	0.003575
1	3.127046	-1.802152	-0.018466
8	1.504873	3.474396	0.348100
1	2.452894	3.668584	0.268675
8	10.230695	-1.397504	0.489418
8	10.049931	-1.320663	-1.657366
9	-7.259969	-1.439931	-2.231167
9	-5.708046	0.793222	1.584654
9	-3.862189	-1.352999	2.989426
9	-4.160031	-1.471992	-1.670545

Table S20: Dimer 2. Cartesian coordinates of the ZZ to Z_PE_F transition state in Å.

7	-4.405081	-1.309361	1.249141
7	-5.412821	-1.038306	0.627281
6	-6.525294	-0.764195	-0.031739
6	-6.914688	-1.475921	-1.177944
6	-7.398979	0.262521	0.361488
6	-8.070555	-1.206375	-1.868417
6	-8.559894	0.554599	-0.311060
6	-8.907788	-0.182849	-1.437727
1	-8.303064	-1.802522	-2.741621
1	-9.182039	1.360955	0.055922
1	-9.819939	0.037815	-1.973928
6	-3.179855	-0.672773	0.885471
6	-2.969372	0.231629	-0.154429
6	-2.069023	-1.004612	1.658628
6	-1.738798	0.794812	-0.430012
6	-0.805787	-0.488286	1.440808
6	-0.659779	0.418065	0.382771
6	0.691817	1.002331	0.112456
6	1.518131	0.387348	-0.837898
6	1.078360	2.157561	0.799989
6	2.777370	0.934720	-1.076586
6	2.335267	2.682571	0.522455
6	3.218280	2.052420	-0.362331
7	4.413012	2.780689	-0.596486
7	5.540888	2.289881	-0.744580
6	5.845429	0.918390	-0.517776
6	5.515521	0.295879	0.684067
6	6.653825	0.277828	-1.453472
6	5.959307	-0.990239	0.931180
6	7.079535	-1.014811	-1.224237
6	6.721816	-1.630280	-0.032893

1	5.720862	-1.493264	1.856996
1	7.689695	-1.539421	-1.944837
6	0.347217	-0.886151	2.320086
1	1.219731	-1.146621	1.720576
1	0.634499	-0.061506	2.976673
1	0.088967	-1.738655	2.944195
6	-1.588857	1.758634	-1.574766
1	-0.901884	2.563548	-1.317033
1	-1.188921	1.252403	-2.457214
1	-2.547768	2.194060	-1.848184
1	4.930021	0.816854	1.429197
1	6.931564	0.796311	-2.361548
7	7.182922	-2.996459	0.223922
6	0.206903	2.872051	1.796704
1	-0.000315	3.893027	1.467442
1	-0.743933	2.363696	1.941157
1	0.704852	2.947320	2.766117
6	1.104589	-0.831400	-1.622642
1	0.142211	-1.214715	-1.292175
1	1.011537	-0.612536	-2.691593
1	1.827082	-1.645467	-1.509168
8	3.605057	0.443948	-2.029642
1	3.182485	-0.272702	-2.515947
8	2.710917	3.819760	1.153944
1	3.547415	4.122335	0.767192
8	6.876504	-3.518128	1.281936
8	7.849621	-3.548905	-0.633746
9	-6.084937	-2.468205	-1.583510
9	-7.042168	0.970906	1.460711
9	-2.247386	-1.872869	2.667078
9	-4.014449	0.570143	-0.928741

Table S21: Dimer 1. Approximate Cartesian coordinates of the EE↔ EZ$_F$ conical intersection in Å.

7	5.430856	0.622686	-0.140490
7	6.180357	-0.363953	-0.185896
6	7.563837	-0.078742	-0.349428
6	8.404315	-1.187442	-0.367042
6	8.092909	1.205690	-0.489553
6	9.773638	-1.019911	-0.521648
1	7.969655	-2.173539	-0.257872
6	9.458847	1.365649	-0.644928
1	7.430303	2.060103	-0.476101
6	10.301913	0.256197	-0.661001
1	10.425881	-1.884381	-0.534075
1	9.873854	2.360280	-0.754879
1	11.369865	0.390970	-0.782818
6	4.046981	0.334937	0.014865
6	3.514812	-0.948845	0.096327
6	3.211954	1.440636	0.084700
6	2.148863	-1.132018	0.247936
1	4.175851	-1.803064	0.039460
6	1.837521	1.289658	0.237295
1	3.650391	2.429352	0.019339
6	1.310588	-0.004912	0.318186
6	-0.160826	-0.192752	0.480688
6	-0.987270	-0.290778	-0.637571
6	-0.725286	-0.271317	1.754570
6	-2.339599	-0.467725	-0.472848
1	-0.584880	-0.228642	-1.640497
6	-2.078891	-0.448376	1.884295
1	-0.114185	-0.197111	2.644888
6	-2.953875	-0.560822	0.789831
7	-4.283915	-0.730429	1.015593

7	-5.192993	-0.977363	0.179052
6	-6.214296	-0.182052	-0.236956
6	-6.372058	1.180435	0.081371
6	-7.208194	-0.722165	-1.072884
6	-7.423102	1.938396	-0.377127
6	-8.269545	0.005019	-1.549445
6	-8.377477	1.345899	-1.195209
1	-7.485077	2.980661	-0.093759
1	-8.999511	-0.474926	-2.187587
1	-9.210367	1.931577	-1.560772
6	0.951042	2.504880	0.312312
1	0.221995	2.516694	-0.500870
1	0.387897	2.529738	1.247653
1	1.543197	3.417419	0.248907
6	1.583408	-2.525545	0.334752
1	1.067953	-2.687107	1.284080
1	0.856454	-2.710076	-0.459117
1	2.375924	-3.268428	0.248852
9	-2.617753	-0.527883	3.110925
9	-3.125979	-0.545653	-1.559524
9	-7.089693	-2.018255	-1.403929
9	-5.437308	1.752897	0.860132

Table S22: Dimer 1. Approximate Cartesian coordinates of the ZZ↔ EZ$_F$ conical intersection in Å.

7	5.247730	-1.487484	-0.197837
7	6.001935	-0.646591	-0.713684
6	6.355354	0.581810	-0.196242
6	6.884303	1.543397	-1.066928
6	6.184914	0.889554	1.163754
6	7.205053	2.800297	-0.587358
1	7.016309	1.289675	-2.111222
6	6.496647	2.158288	1.622909
1	5.793990	0.132898	1.832650
6	7.013030	3.115315	0.755457
1	7.600174	3.545879	-1.266809
1	6.352392	2.398241	2.669708
1	7.265368	4.102290	1.122736
6	3.868982	-1.466625	-0.180087
6	3.132075	-0.659836	-1.056214
6	3.202472	-2.268159	0.749691
6	1.747764	-0.614858	-0.977249
1	3.658037	-0.056122	-1.785848
6	1.819544	-2.245842	0.836933
1	3.785642	-2.893775	1.414130
6	1.090806	-1.415530	-0.030600
6	-0.399335	-1.386303	0.052831
6	-1.038786	-0.559720	0.972153
6	-1.164486	-2.192548	-0.788370
6	-2.415439	-0.539046	1.022405
1	-0.478070	0.068768	1.651750
6	-2.535599	-2.154403	-0.692808
1	-0.701922	-2.850134	-1.512707
6	-3.209948	-1.316773	0.187445
7	-4.624836	-1.449181	0.318899

7	-5.400366	-0.486123	0.305601
6	-4.989590	0.847771	0.004468
6	-4.360839	1.220050	-1.179512
6	-5.406745	1.880215	0.837355
6	-4.127431	2.536716	-1.513808
6	-5.178879	3.206922	0.553182
6	-4.534983	3.529275	-0.634707
1	-3.638562	2.767746	-2.450625
1	-5.505027	3.966107	1.251036
1	-4.353151	4.567349	-0.879499
6	1.123850	-3.100993	1.864007
1	0.616193	-2.487811	2.612038
1	0.365864	-3.738254	1.404246
1	1.838719	-3.740192	2.381296
6	0.976158	0.273596	-1.918099
1	0.331771	-0.311358	-2.578112
1	0.331792	0.965886	-1.372183
1	1.654505	0.857787	-2.539146
9	-3.272894	-2.937054	-1.497925
9	-3.019449	0.240628	1.937973
9	-6.034828	1.545895	1.976982
9	-3.999342	0.253335	-2.043467

Table S23: Dimer 1. Approximate Cartesian coordinates of the ZZ↔ ZE$_F$ conical intersection in Å.

7	-5.905111	-1.610841	-0.584647
7	-6.770241	-0.729567	-0.537660
6	-6.463524	0.666385	-0.393197
6	-7.156425	1.367752	0.587048
6	-5.639175	1.336407	-1.292158
6	-6.966747	2.735664	0.715610
1	-7.828187	0.834162	1.248605
6	-5.481695	2.710003	-1.176847
1	-5.134489	0.791808	-2.079470
6	-6.131018	3.410028	-0.166815
1	-7.486270	3.278567	1.495723
1	-4.846775	3.235248	-1.879955
1	-5.997261	4.481021	-0.076797
6	-4.511751	-1.354826	-0.348263
6	-4.067728	-0.778005	0.832733
6	-3.605962	-1.855017	-1.271039
6	-2.705517	-0.656440	1.082826
1	-4.781289	-0.426978	1.567433
6	-2.238828	-1.720768	-1.059794
1	-3.973744	-2.340684	-2.167309
6	-1.792258	-1.117296	0.124415
6	-0.327528	-0.974759	0.374207
6	0.345191	0.177627	-0.027040
6	0.385238	-1.993078	1.008029
6	1.694273	0.298652	0.207993
1	-0.172685	0.983258	-0.531270
6	1.730385	-1.838770	1.230764
1	-0.102230	-2.903881	1.330763
6	2.445077	-0.692935	0.856119
7	3.779551	-0.611836	1.147929

7	4.516459	0.402135	1.037524
6	5.523009	0.591814	0.128909
6	5.783645	-0.231950	-0.978089
6	6.393223	1.678622	0.297042
6	6.831616	-0.007129	-1.840357
6	7.450386	1.931203	-0.541372
6	7.668609	1.078959	-1.618147
1	6.978756	-0.677200	-2.676805
1	8.087565	2.784385	-0.351548
1	8.494826	1.266126	-2.290751
6	-1.268414	-2.231647	-2.091961
1	-0.616974	-1.432828	-2.452929
1	-0.622326	-3.009910	-1.680064
1	-1.799165	-2.649319	-2.947019
6	-2.236921	-0.034369	2.371977
1	-1.592606	-0.716257	2.930905
1	-1.658224	0.873361	2.186839
1	-3.084296	0.228105	3.004718
9	2.414505	-2.817169	1.846172
9	2.336007	1.400043	-0.218845
9	6.168252	2.489652	1.344059
9	4.953216	-1.264191	-1.210125

Table S24: Dimer 2. Approximate Cartesian coordinates of the EE↔ Z_PE_F conical intersection in Å.

7	-5.111378	-0.715097	0.306024
7	-5.979925	0.084198	-0.083836
6	-7.297871	-0.417968	-0.089387
6	-8.331550	0.506295	0.070390
6	-7.688382	-1.740501	-0.323549
6	-9.664139	0.162650	0.041174
6	-9.012112	-2.121427	-0.378543
6	-9.998415	-1.165059	-0.185041
1	-10.415857	0.925521	0.191335
1	-9.254096	-3.157360	-0.574566
1	-11.039822	-1.457223	-0.217115
6	-3.781254	-0.258583	0.213220
6	-3.262825	0.601607	-0.751395
6	-2.864230	-0.793541	1.115809
6	-1.921918	0.939818	-0.825726
6	-1.516130	-0.495883	1.113299
6	-1.055167	0.384903	0.122882
6	0.402034	0.725520	0.078772
6	1.250514	-0.018561	-0.751992
6	0.883364	1.777814	0.871745
6	2.604448	0.302091	-0.782020
6	2.238001	2.079222	0.816936
6	3.123693	1.364743	-0.014695
7	4.435863	1.738625	-0.028965
7	5.358667	1.445138	-0.842932
6	6.410640	0.612028	-0.520563
6	6.529572	-0.014485	0.733815
6	7.404106	0.411780	-1.494777
6	7.611285	-0.821086	1.000695
6	8.481737	-0.400006	-1.231545

6	8.579933	-1.008354	0.018149
1	7.718282	-1.306823	1.959912
1	9.248772	-0.567221	-1.973719
6	-0.601533	-1.101763	2.141957
1	0.329515	-1.437055	1.685408
1	-0.344681	-0.370978	2.912923
1	-1.072380	-1.951180	2.632165
6	-1.458213	1.862130	-1.919810
1	-0.512105	2.332422	-1.659906
1	-1.315071	1.313958	-2.855059
1	-2.194339	2.642651	-2.109980
1	5.769249	0.158003	1.484676
1	7.302813	0.902758	-2.454170
7	9.716830	-1.855476	0.302210
6	-0.004936	2.609358	1.761640
1	0.020804	3.666976	1.479033
1	-1.043007	2.292676	1.700857
1	0.294054	2.536033	2.812277
6	0.770936	-1.169309	-1.599030
1	1.268455	-2.104961	-1.323215
1	-0.297329	-1.333400	-1.485630
1	0.957329	-0.991102	-2.662935
8	9.794876	-2.370238	1.408743
8	10.555135	-2.011738	-0.574102
8	2.791736	3.079951	1.541245
1	2.124162	3.524564	2.074805
8	3.495721	-0.382722	-1.535331
1	3.052906	-1.061525	-2.057162
9	-7.999998	1.790350	0.282773
9	-6.755896	-2.675949	-0.556042
9	-4.083061	1.094048	-1.696040
9	-3.341860	-1.635233	2.053049

Table S25: Dimer 2. Approximate Cartesian coordinates of the ZZ↔ E_PZ_F conical intersection in Å.

7	5.015365	-1.885821	1.306176
7	6.104065	-1.570102	0.814385
6	6.217323	-0.641187	-0.264129
6	7.131394	0.401318	-0.161299
6	5.616754	-0.821283	-1.505213
6	7.405749	1.258579	-1.202144
6	5.874646	0.001024	-2.581005
6	6.768341	1.049537	-2.418618
1	8.107348	2.068653	-1.055992
1	5.381871	-0.190126	-3.524721
1	6.975878	1.708726	-3.251081
6	3.799846	-1.219169	0.967074
6	3.591225	0.137103	1.152693
6	2.688965	-1.979086	0.626839
6	2.361806	0.751079	0.981388
6	1.434877	-1.442558	0.419030
6	1.285549	-0.057879	0.603732
6	-0.062392	0.556635	0.388009
6	-0.990294	0.565434	1.436962
6	-0.343565	1.115709	-0.866500
6	-2.228581	1.157685	1.214254
6	-1.586125	1.700556	-1.062111
6	-2.555139	1.701667	-0.042816
7	-3.751343	2.302025	-0.295933
7	-4.901478	2.229195	0.197842
6	-5.786986	1.183057	-0.043851
6	-5.651679	0.283457	-1.113821
6	-6.878507	1.056408	0.832588
6	-6.574008	-0.726219	-1.287312
6	-7.797103	0.045887	0.663368
6	-7.632932	-0.840243	-0.396309

1	-6.484863	-1.421768	-2.109327
1	-8.634346	-0.066635	1.336873
6	0.286297	-2.321290	0.006342
1	-0.533804	-2.255708	0.723049
1	-0.105220	-2.013431	-0.965535
1	0.594327	-3.361882	-0.064809
6	2.240989	2.227855	1.240128
1	1.346944	2.637331	0.775325
1	2.181355	2.426164	2.313596
1	3.109875	2.763320	0.857725
1	-4.829606	0.380827	-1.807581
1	-6.977189	1.760986	1.647608
7	-8.603047	-1.907605	-0.585172
6	0.666596	1.120294	-1.983524
1	1.421394	1.896947	-1.829905
1	1.190257	0.166141	-2.048213
1	0.185112	1.312989	-2.940083
6	-0.704089	-0.011448	2.798920
1	0.279834	-0.472130	2.840357
1	-0.729691	0.760359	3.574631
1	-1.433244	-0.781842	3.069512
8	-3.187854	1.238492	2.166232
1	-2.884482	0.843453	2.991325
8	-1.868396	2.270769	-2.258905
1	-2.740578	2.684400	-2.221008
8	-8.465763	-2.653923	-1.542153
8	-9.516529	-2.009155	0.218953
9	7.742956	0.579583	1.021899
9	4.780788	-1.864080	-1.664570
9	2.883123	-3.303907	0.468662
9	4.634074	0.887559	1.567130
